# The Potential Role of Hydrogen in Decarbonization: Exploring Global Supply Chain Impacts and Hydrogen Use in the United Kingdom

**DOI:** 10.1021/acs.est.4c14368

**Published:** 2025-07-15

**Authors:** Alice Bennett, André Cabrera Serrenho

**Affiliations:** Department of Engineering, 2152 University of Cambridge, Trumpington Street, Cambridge CB2 1PZ, United Kingdom

**Keywords:** hydrogen imports, global supply chains, emissions, decarbonization

## Abstract

Decarbonization of all sectors is needed to mitigate the impacts of climate change. To accomplish this, hydrogen use has been suggested in many industries that currently rely on fossil fuels. Yet, the emissions intensity of hydrogen depends on how it is produced and distributed. Additionally, it is unclear whether hydrogen use leads to a reduction in GHG emissions compared to alternative decarbonization options such as electrification with renewables. Here, we systematically compare the decarbonisation potential of supplying hydrogen to the United Kingdom from a wide range of global supply chains. We do this by assessing 37,000 configurations of the hydrogen supply chain from primary energy production through to end-use. We find that imports of green hydrogen production are unlikely to be compatible with the UK Low Carbon Hydrogen Standard. The maximum mitigation potential is achieved when electrification is prioritized, and hydrogen used only for applications where electrification is not viable. This leads to a reduction of up to 280 Mt CO_2_e/a across all sectors considered in the UK. In the short term, use of domestic green hydrogen infrastructure should focus on displacing existing gray hydrogen use.

## Graphical abstract

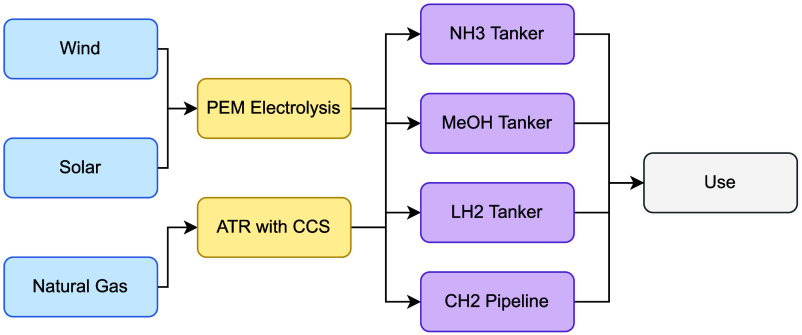

## Introduction

Meeting global climate targets will require the decarbonization of all energy uses. Hydrogen has a role to play in this transition, particularly in sectors where electrification may not be feasible such as aviation, shipping and high temperature industrial heat.^1,2^ As such, the International Energy Agency^3^ expects that global demand for hydrogen will increase from 120 Mt/year today to 500–800 Mt/year by 2050. However, it is unclear whether future demand can align with climate targets. Current hydrogen production relies on fossil fuels, resulting in over 2% of global greenhouse emissions,^3^ and therefore new low carbon supply chains are required.

Two production methods are commonly considered in national hydrogen strategies: renewable electrolytic production (green hydrogen) and fossil fuel reforming with carbon capture and storage (blue hydrogen).^4^ Green hydrogen has been shown to be able to align with climate targets.^5^ However, although earlier work suggested that blue hydrogen could be competitive with green hydrogen in specific circumstances,^6^ other work has suggested this is unlikely due to the impacts of upstream methane and hydrogen emissions.^7,8^ Therefore, to maximize use of green hydrogen while minimizing costs, national strategies include the use of imported green hydrogen from areas with favorable renewable energy potential such as South America.^9,10^

However, hydrogen use is unlikely to be the optimal decarbonization pathway for all proposed applications, as in some cases alternative options exist such as direct electrification. In the “Clean Hydrogen Ladder”, Liebreich et al. summarize which applications they believe will be more easily decarbonised by other routes.^11^ For example, they suggest that use of hydrogen for domestic heating is “Uncompetitive” as heat pump use is likely to result in lower emissions.^12^ Avoiding these uncompetitive uses could substantially reduce total demand for hydrogen. This is demonstrated by Vatankhah Ghadim et al., who found that the hydrogen demand in New Zealand would reduce by 35% if “Uncompetitive” uses, such as domestic heating, are electrified instead.^13^ Despite this, sectors labeled as uncompetitive have still seen investments from both private companies and governments due to uncertainty about the optimal pathways.^14,15^

The potential impact of investing in “Uncompetitive” hydrogen applications is unclear. Few studies have compared the potential reduction in greenhouse gas emissions from hydrogen use to alternative decarbonization options. For example, though Pivac et al. found that deploying hydrogen fuel cell vehicles (FCEVs) should be high priority,^16^ they did not consider use of battery electric vehicles (BEVs). Other studies have shown that use of BEVs can lead to less emissions than FCEVs on a tank-to wheel basis,^17,18^ though some uncertainty remains for heavy duty vehicles due to the embodied emissions.^19^ These discrepancies between results show that the comparison and acknowledgment of alternative decarbonization options is important.

Overlooking hydrogen supply chain impacts may also result in generous assessments of the role hydrogen may have, especially if global trade is considered. Though imports of renewable electrolytic hydrogen have been shown to align with targets set for low emission hydrogen,^20–23^ it was highlighted by de Kleijne et al. that the impacts of embodied emissions, and hydrogen leakage are often overlooked.^24^ To increase understanding of the emissions intensity of global hydrogen production, de Kleijne et al. conducted a life cycle analysis of 1000 planned renewable electrolysis projects, including consideration of these impacts.^24^ They found that the emissions intensity could range from 0.3–36.5 kg CO_2_e/kg H_2_. As the upper limit is 15 times larger than the UK Low Carbon Hydrogen Standard (2.4 kg CO_2_e/kg H2),^23^ this shows that some renewable electrolysis pathways could exceed carbon targets.

One of the key factors determining the emissions intensity of global hydrogen supply chains is shown to be offshore transmission.^25^ Studies comparing different hydrogen carriers have found that the optimal choice depends on the distance traveled, as there are trade-offs between impacts such as conversion losses and fuel use.^26,27^ The planned end-use may also impact which carrier is optimal, since further energy use and losses from reconversion can be avoided in some cases. For example, if imported ammonia is used directly for fertilizer production, the emissions intensity can be comparable to use of ammonia produced locally.^28^ Equivalent results may exist for other scenarios, with some global supply chains optimal for specific end-uses.

Existing literature has shown that while some hydrogen supply chains could have lower emissions intensities than current processes, others may not.^24^ It has also been shown that for some applications alternative decarbonization options should be prioritised.^17^ However, analysis of the emissions intensity of hydrogen use compared to alternative decarbonization options considering a wide range of supply chains has not been found. Filling this gap is the purpose of this study and would allow the impact of supply chain choice to be revealed, as well as guiding prioritization of hydrogen uses for decarbonization.

To fill this gap, the open-source model presented by Bennett and Serrenho^5^ is extended to include an analysis of global hydrogen supply chains and hydrogen end-use applications. In the extended model, hydrogen production methods, transmission methods, and end-uses are linked using an algorithm to find the possible supply chains and calculate the energy and emissions intensity. The model explores potential hydrogen uses in the UK, served by global production from the largest planned green hydrogen production facilities.

## Method

To assess the impact of global hydrogen imports on the abatement potential of hydrogen use in the UK, this work extends a model which was previously built by the authors to explore the energy and emissions intensities of onshore and offshore hydrogen production in the UK.^5^ As the previous model only explored domestic production, in this work it has been extended to enable analysis of international supply chains. Additionally, the previous model did not include the end-uses of hydrogen within the system boundary. This was added to the model in this work in order to be able to estimate the reduction in GHG emissions possible from hydrogen use in the UK. A similar approach to Vatankhah Ghadim et al.^13^ was used for this component, with current demands for each sector found from either governmental reports, industry data, or academic work. This modeling approach was chosen over energy systems optimization modeling, or integrated assessment modeling, as use of these tools may not have allowed detailed assessment of the impact of each supply chain.

### System Boundary

The system boundary includes all stages of hydrogen supply chains from primary energy generation through to end use application with transmission, storage, and conversion considered. A simplified version of the system boundary and model scope is shown in Figure 1. This is a significant expansion of the previous work which only considered primary energy production, hydrogen production, and transmission from offshore production locations.

**Figure 1:**
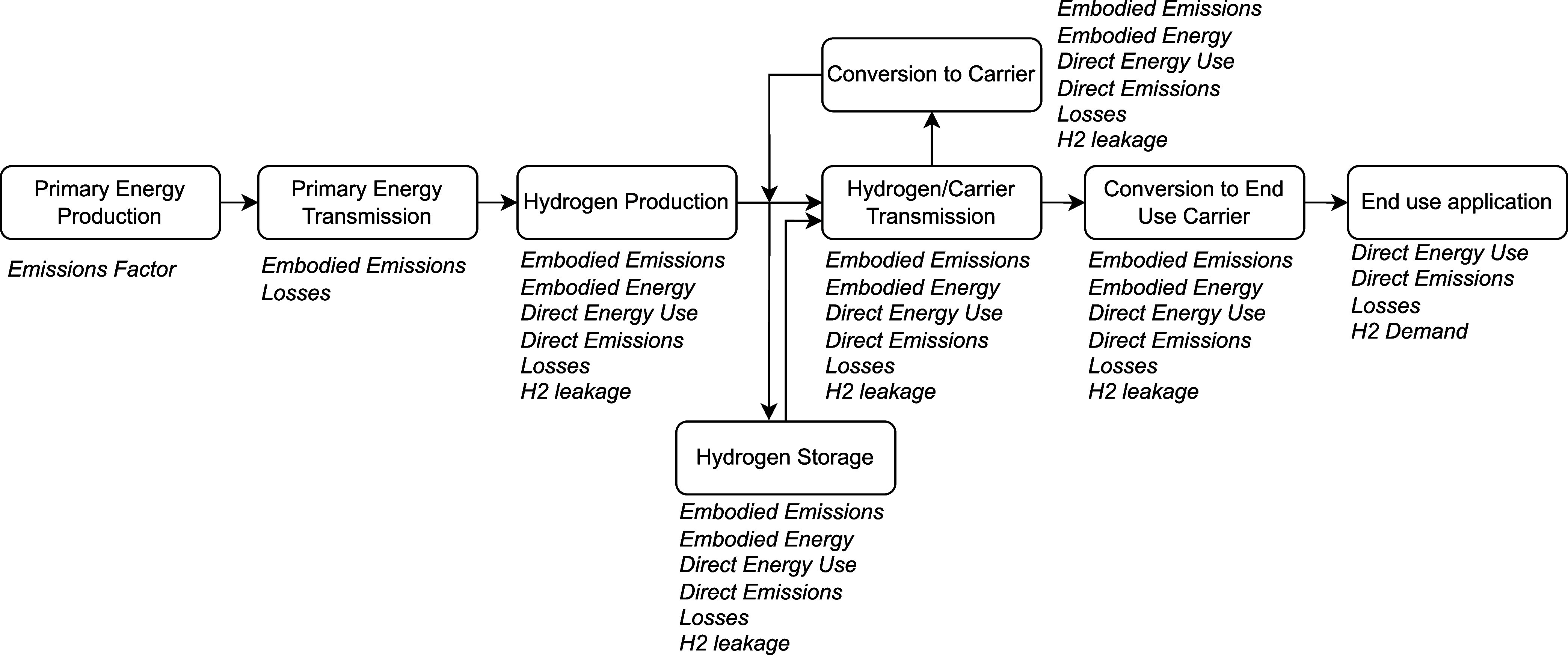
Model scope and key inputs considered for each stage of the supply chain.

Within each of the components of the system multiple technology options have been assessed. In order to maintain a uniform analysis boundary, where possible, primary inventory data was used to define the embodied and direct energy and emissions intensity of each of the processes. When primary data was not available, previously conducted LCAs were used to estimate the emissions intensity which in some cases had a different system boundary. This was an unavoidable limitation given the number of processes considered. It was decided to focus on current emissions factors and energy uses for ancillary infrastructure, such as shipping, because hydrogen use is not only a far future option and deployment of technology has begun for some sectors. The emissions intensity of processes was defined in this work using the 100 yr GWP of greenhouse gases. The indirect impacts of hydrogen leakage throughout the supply chain were also included within the assessed boundary as seen in Figure 1.

### Supply Chain Definition

To estimate the energy and emissions intensity of potential hydrogen supply chains, the possible supply chains needed to be defined. As shown in Figure 1, each of the supply chains within the scope consist of stages such as primary energy generation, hydrogen production, transmission, storage, and conversion. The algorithm used to define the supply chains is detailed in section 1.2 of the SI and is based on both physical compatibility and logical arguments. For example, it has been decided to restrict the possible options to prevent both ammonia and methanol from being used as carriers within the same supply chain. Otherwise, additional stages of conversion and reconversion would be required which would decrease the efficiency and lead to higher costs.^9^

As all options are systematically considered so that the optimal supply chains can be found, the number of conversion stages differs depending on the supply chain. Several conversion steps would be required if either ammonia or methanol is used as an intermediate carrier and hydrogen is required for the end use application. Conversely, reconversion could be avoided in cases of direct ammonia or methanol use.

### Calculations

Once the supply chains have been defined, the specific energy and emissions intensity of each stage, prior to use, are calculated. They are calculated by combining the embodied energy and emissions due to construction and material use and the direct energy uses and emissions (process impacts). This includes the impacts of conversion from the transmission vector to hydrogen at the final stage, if required.

In order to combine the values, they need to be adjusted to the same units (kWh/kgH_2_ or kg CO_2_e/kgH_2_). An example of this adjustment is shown in eq 1 for the embodied energy where *E*_*e,x,f*_, *E*_*e,x,d*_ are the fixed and dynamic embodied energies (kWh, kWh/km) of stage *x*, *L*_*x*_ is the lifetime of infrastructure (years), *C*_*x*_ is the maximum capacity of production (kg H_2_/year), *CF*_*x*_ is the capacity factor of production, and *d*_*x*_, is the distance traveled in the stage (km).

1 $$E_{\mathrm{emb}}=\frac{\sum E_{e,x,f}+d_{x}\sum E_{e,x,d}}{L_{x}C_{x}CF_{x}}$$

The total energy of the stage is then found by combining the embodied, *E*_emb_, maintenance, *E*_O&M_, and process, *E*_pro_, energy intensities as shown in eq 2.

2 $$E_{\mathrm{stage},x}=E_{\mathrm{emb}}+E_{O\&M}+E_{\mathrm{pro}}$$

The total energy intensity of hydrogen production and transmission can then be calculated, *E*_upstream_, as shown in eq 3 for international supply chains, where *c*_*x*_ is the stage yield coefficient and accounts for losses that occur through the supply chain. Similar equations are used to calculate the emissions intensity of hydrogen production and transmission, but additionally the emissions intensity of energy use is accounted for. For further details see section 1 of the Supporting Information (SI).

3 $$E_{upstream}=\sum_{2}^{10}E_{x}c_{x}$$

At this stage the calculations have not included the impacts due to upstream energy production and transmission, which also need to be accounted for to find the energy required for hydrogen supply, *E*_*SC*_, and the emissions intensity of hydrogen supply, *G*_*SC*_. In this work only the losses from natural gas and electricity transmission are considered, *n*, leading to eqs 4 and 5 where *EF*_*i*_ is the emissions factor of energy source *i*.

4 $$E_{\mathrm{SC}}=nE_{\mathrm{upstream}}$$

5 $$G_{\mathrm{SC}}=nE_{\mathrm{upstream}}EF_{i}+G_{\mathrm{upstream}}$$

Once the emissions intensity and energy intensity of hydrogen supply and transmission are calculated they are combined with the use assumptions to calculate the impacts of the whole supply chain. This consists of three steps: hydrogen is converted from the final transmission vector to the vector specified for use if different (hydrogen, ammonia, or methanol), the final energy intensity is calculated including energy demand at the point of use, and then the total emissions intensity is found including direct emissions from the use process.

The energy and emissions intensity of converting the hydrogen to the form required, *E*_U0_ and *G*_U0_, are calculated using the same method as the impacts of upstream stages. The total energy intensity of hydrogen production, *E*_T_, is then found using eq 6, where *n*_U0_ is the yield of conversion to the final hydrogen carrier. Similarly, the emissions intensity,*G*_*T*_, is given in eq 7.

6 $$E_{T}=\left(E_{U0}+E_{\mathrm{SC}}/n_{U0}\right)$$

7 $$G_{T}=\left(G_{U0}+G_{\mathrm{SC}}/n_{U0}\right)$$

To estimate the impacts of hydrogen use, each use case is defined in terms of the amount hydrogen (or hydrogen carrier) required per unit of activity (kg H_2_ equiv/unit), *A*. (The units are specified for each process in the SI). The energy intensity and emissions intensity of hydrogen use in the process can then be calculated and added to other energy inputs and emissions that occur *E*_O_, *G*_O_ (kWh/unit and kg CO_2_e/unit respectively) to result in the total energy and emissions intensity of hydrogen use.

8 $$E=A\left(E_{T}\right)+E_{O}$$

9 $$G=A\left(G_{T}\right)+G_{O}$$

These results are then compared to the energy and emissions intensity of current processes and alternative decarbonization options.

### Electrification Assumptions

To compare the use of hydrogen to electrification, and show how this impacts decarbonization potential, we completed an analysis of the emissions intensity of electrifying processes. However, as most emissions from aviation, shipping, high temperature heat, and fertilizer production cannot be avoided by direct electrification they were excluded from this part of the analysis. In these cases, other decarbonization pathways such as use of biofuels, are possible but were not considered in this work.

The emissions intensity of electrification was calculated based on the energy demand and remaining direct emission from the electrified process but did not include assessment of the wider supply chain. Two scenarios were considered for the emissions intensity of electricity: renewable energy (15 g CO_2_e/kWh) and current grid electricity (160 g CO_2_e/kWh).

### Uncertainty Analysis

There is a large degree of uncertainty in some of the inputs required, such as the embodied emissions of infrastructure yet to be built, and the emissions intensity of future electricity production. To address this, a Monte Carlo analysis has been used to find possible upper and lower bounds of the impacts. Each of the input variables are assigned lower and upper bounds based on literature, analogies with other systems, or physical limits. A value within the bound is selected at random for each of the 2500 iterations. Additionally, global sensitivity analysis is conducted using the Sobol method to assess the importance of the uncertainty in variable groups on the results. Further details of the method and assigned distributions can be found in section 1 and 2 of the SI.

## Case Study Scope

The model described above can be used to explore the energy and emissions intensity of supplying hydrogen to any country. In this work, the model is applied to the United Kingdom. The following sections describe how the primary energy sources, production locations, production methods, transmission options, use location and end use applications were chosen. Further details can be found in section 2 of the SI.

### Primary Energy Sources

The primary energy sources included in the model are natural gas, wind power and solar power. The emissions factor has been specified for each country as it has been shown to vary depending on the technology used and natural resources available. In the case of natural gas, the emissions factors were obtained from published literature and include the impacts of upstream methane emissions. For renewable energy sources, the utilization factor is also included to account for the impact of intermittency on the utilization rate of an electrolyzer. This is based on work by Mendler et al. which found the most economical ratio between electrolyzer and renewable capacity based on renewable energy generation patterns.^29^ Other primary energy sources such as nuclear energy and biomass were not included within the scope due to availability^30^ and relevance as planned projects are predominately limited to use of natural gas with CCS or wind/solar power.^31^

### Production and Use Locations

The production locations included in the case study were chosen based on the largest planned projects contained within the IEA Hydrogen Project Database.^31^ The projects chosen are locating in Australia, Mauritania, Egypt, Kazakhstan, Spain, UK, Brazil and USA. The total transmission distances to use range from 0 km (UK) to more than 10,000 km (Australia), encompassing most possible ranges. Additional production locations that may become relevant to consider in the future, such as Chile or China, can also be explored using the open access model presented here.

The location of the end uses is assumed to be Teesside in the UK, which is colocated with domestic production. This represents a best-case scenario for domestic hydrogen as minimal transmission is required from production to use. Further domestic transmission will reduce the benefits of hydrogen use and could be explored in greater detail once the most optimal supply chains are known.

### Production Methods

The use of Polymer Electrolyte Membrane (PEM) electrolysis is modeled for each location, while Autothermal Reforming with CCS (ATR with CCS) is also modeled for some locations with access to natural gas extraction. Other fossil-fuel based production methods such as Steam Methane Reforming with CCS were not considered as previous work has shown that they would result in higher emissions than ATR with CCS.^5^ This includes the UK, Egypt, Mauritania, Kazakhstan, and Brazil. Key production method specifications are shown in Table 1.

**Table 1:** Key Production Method Properties

	**electricity demand (kWh/kg)**	**natural gas demand (kWh/kg)**	**water demand (kg/kg H**_**2**_ **)**	**carbon capture rate**	**refs**
**PEM electrolysis**	46.6–62.8	0	8.9	NA	^32^
**ATR with CCS**	3.6–4.1	41.7–49.0	0	90%	^33^

### Transmission Methods

Based on previous literature and planned projects, four hydrogen carriers have been included in the scope of the case study. These are compressed hydrogen, liquified hydrogen, ammonia, and methanol. The performance assumptions of conversion and reconversion for each of the carriers are listed in section 2.5 of the SI. Notably we do not account for negative emissions from carbon capture during methanol synthesis to avoid double counting. However, carbon capture is included within the scope of methanol reforming. Electricity and natural gas are also included as energy carriers. To transport the energy and hydrogen carriers four types of infrastructure are modeled. These are pipelines (onshore and offshore), cables (onshore and offshore), trucks and tanker ships. The carrying capacity of transmission infrastructure has been adjusted based on the properties of the carrier as detailed in section 2.6 of the SI. The infrastructure available to transport the carrier depends on both the type of journey (onshore or offshore) and the length, for example offshore pipeline length is limited to 1,200 km based on the length of existing natural gas pipelines.

### End Use Applications

The hydrogen uses included within the case study have been taken from the Clean Hydrogen Ladder.^11^ However, based on the method described by Vatankhah Ghadim et al.^13^ some uses not been included due to their relevance and magnitude. Other applications have been combined due to similarity of the use cases. For example, use of hydrogen fueled taxis has been merged with use of hydrogen fueled cars. In total 28 of the 35 applications from the hydrogen ladder are included, as shown in Table 2. The assumptions made for each use case are detailed in section 2.8. of the SI.

**Table 2:** Included Hydrogen Applications and Hydrogen Use Type

**hydrogen use**	**applications**					
**fuel cell**	GRID balancing	trucks	vans	cars	motorcycles	buses
**combustion**	high temp heat	domestic heat	mid/low temp heat	commercial heat	aviation	
**via ammonia**	fertiliser production	shipping (combustion)				
**other chemical process**	steel (H2 DRI)	biogas upgrading	methanol production			

Demand for each application is based on latest demand profiles for the applications from either governmental reports, industry data, or academic work. This does not account for changes in demand for some services due to population growth or other changes but demonstrates the current potential for hydrogen use in the UK.

## Results

1960 unique supply chains are found by combining the energy sources, production methods, production locations, transmission methods and storage options included in the case study scope. These are then combined with the 19 end-uses leading to 37,000 hydrogen supply chain configurations from primary energy source through to end-use application. To begin to understand whether imported hydrogen can align with climate targets, and the role of hydrogen in decarbonization across different sectors, the energy and emissions intensity of the supply chains are explored in the following section. This includes the discussion of the impact of supply chain configuration. The potential emissions abatement of hydrogen use in the UK is explored for various applications, with comparison to direct electrification where suitable.

### Emissions Intensity of Global Hydrogen Supply Chains

Figure 2 reveals the emissions intensity of the supply chain with the lowest impact for each country, production method, and main transmission vector (defined as the carrier used for transmission between the production country and the UK). This figure shows that that while domestic production via electrolysis can align with the UK Low Carbon Hydrogen Standard, most international supply chains are unlikely to. If imports are necessary, the results suggest that transmission distance should be minimized but that the emissions intensity of local energy production is also important to consider. In some cases imports from countries further away can be lower impact, such as Brazil compared to Egypt. Though it might be assumed that energy use in shipping contributes significantly, in section 3.3. of the SI it is shown that use of decarbonised shipping would have limited impact as factors such as increased losses remain.

**Figure 2:**
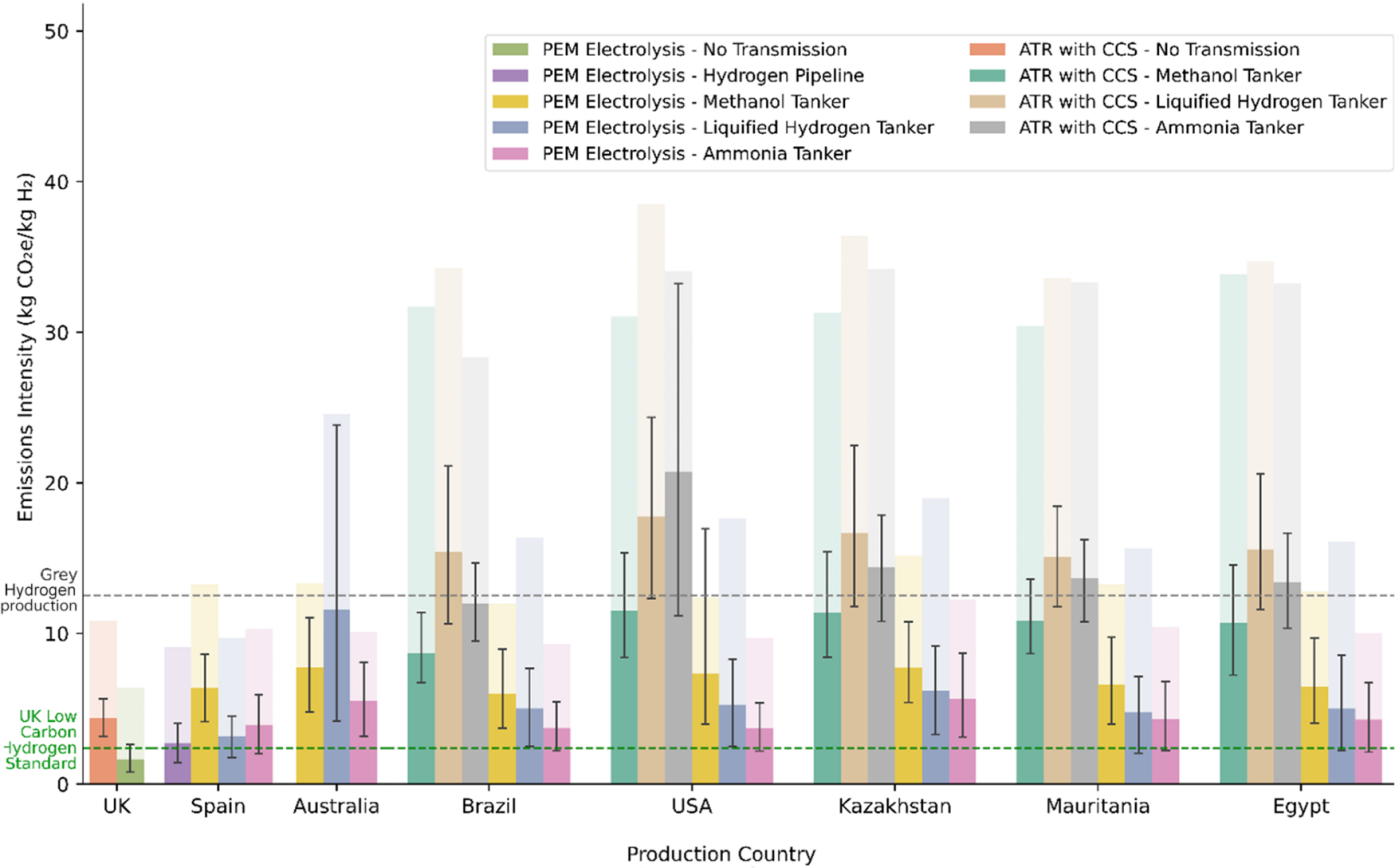
Emissions intensity of both domestic and imported hydrogen supply chains for the lowest emission supply chain for each production country, main transmission vector, and production method. The uncertainty bars show the maximum and minimum bounds of the Monte Carlo analysis for the optimal supply chains. The translucent bars show the emissions intensity of the supply chain with the highest emissions that has the same origin, production method, and main transmission vector. The emissions intensities are compared to the emissions intensity of current gray hydrogen production (12.5 kg CO_2_e/kg H_2_) and the UK Low Carbon Hydrogen Standard (2.4 kg CO_2_e/kg H_2_), which are shown as horizontal dashed lines.

For countries other than Spain (where hydrogen pipelines may be possible), electrolytic hydrogen production combined with either ammonia or liquified hydrogen as the carrier is found to result in the lowest emissions, assuming reconversion to hydrogen is required. For cases where ammonia or methanol are the desired product, equivalent figures are discussed in section 3.1. and 3.2. of the SI.

If, instead of electrolysis, autothermal reforming with CCS (ATR with CCS) is used as the production method, no supply chains were found likely to align with the UK Low Carbon Hydrogen Standard. In many cases, supply chains were instead shown to exceed the emissions intensity of current hydrogen production. In the worst cases the emissions intensity could be increased by over five times compared to current processes.

### Impact of Supply Chain Configuration

As well as understanding which supply chains could lead to the lowest emissions, it is important to discover what impact options such as hydrogen carrier choice and primary energy source have on the emissions intensity. Figure 3 demonstrates that in the UK offshore wind could be used instead of onshore wind power with minimal impact, but solar power is found likely to be significantly worse due to the relatively low solar potential and the impact this has on utilization across the supply chain. Requiring conversion to another hydrogen carrier for transmission or storage would also lead to at least 150% greater emissions in the case of ammonia, and 360% if methanol is used.

**Figure 3:**
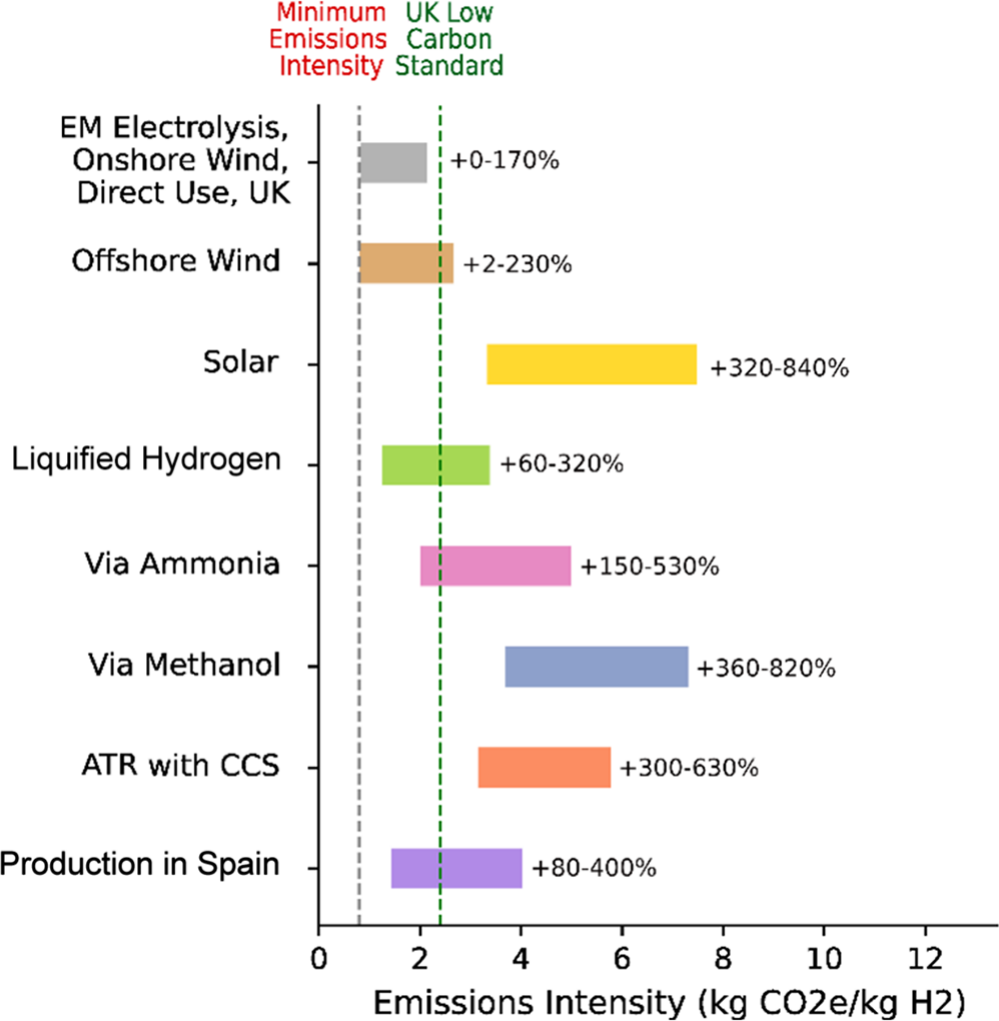
Emissions intensity of the hydrogen supply chain with the lowest possible emissions for delivery of hydrogen, compared to the range of emissions intensities found if a component of the supply chain is substituted for an alternative option. The percentages show the increase compared to the minimum emissions intensity (0.8 kg CO_2_e/kg H_2_). For example, if onshore wind power is substituted with solar power the emissions intensity is found to increase by 320–840% compared to the minimum value. The emissions intensity bars are also compared to the emissions intensity of current gray hydrogen production (12.5 kg CO_2_e/kg H_2_) and the UK Low Carbon Hydrogen Standard (2.4 kg CO_2_e/kg H_2_), which are represented as vertical dashed lines.

Figure 3 also demonstrates the sensitivity of the results to the uncertainty in assumptions by displaying the range of possible values found in the Monte Carlo analysis. From this we can see that, in some cases, the supply chain configuration may be less important than ensuring that the optimal production conditions are possible in some cases. For example, the range found for the emissions intensity of PEM electrolysis powered by onshore wind in the UK (0.8–2.2 kg CO_2_e/kg H_2_) suggests that in some circumstances it could lead to higher emissions than production in Spain (1.5–4.0 kg CO_2_e/kg H_2_). Sobol indices analysis, presented in section 3.4. of the SI, indicated the energy source and production method assumptions have the largest impact on this variability for domestic pathways, with offshore transmission and storage impacts also important for international supply chains.

### The Potential Emissions Abatement from Hydrogen Use in the UK

The sectors included within the model scope, based on the hydrogen ladder by Liebreich et al.,^11^ are found to be responsible for 290 Mt CO_2_e/a in the UK. This includes contributions from all sectors listed in Table 2 in the methods section, such as steel production, aviation, and heating. In Figure 4 it is shown that if all processes were switched to hydrogen, demand for hydrogen would total 28 Mt H_2_/a (■) – more than 30 times current use.^34^ If this was supplied from the lowest emission domestic supply chain, it is found that hydrogen use could abate up to 270 Mt CO_2_e/a. If instead imported hydrogen is used the maximum potential abatement decreases to between 70–230 Mt CO_2_e/a.

**Figure 4:**
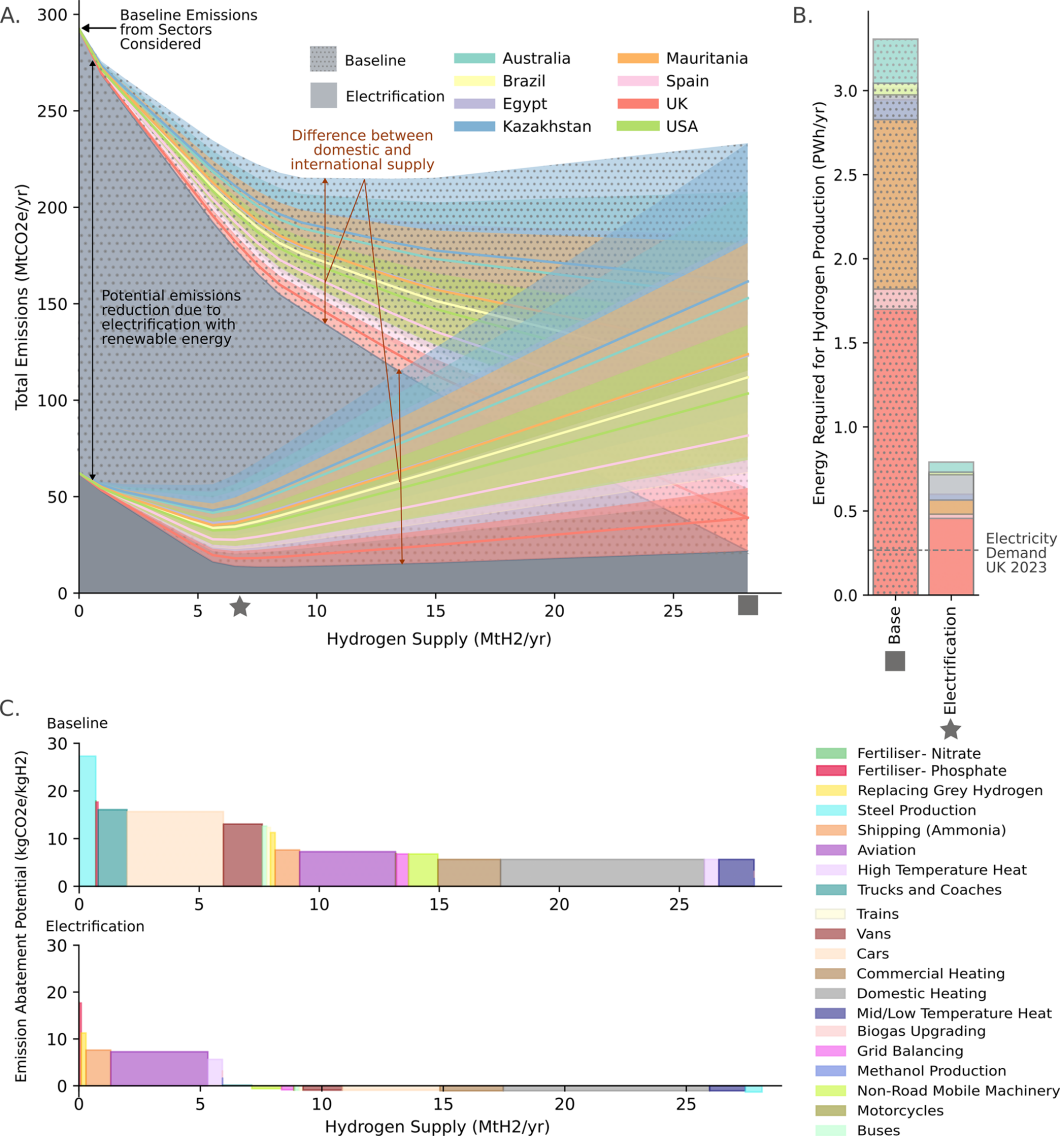
(A) Total emissions in the UK from the sectors considered as a function of the amount of hydrogen available per year, assuming that the minimum emission hydrogen supply chain for each production country is used. The baseline scenario compares the hydrogen supply chain emissions to current emissions whereas in the electrification scenario all viable applications are first electrified using renewable power (assuming 15 g CO2e/kWh). The colored regions encompass the minimum and maximum abatement potential found in the Monte Carlo analysis, given the use of the optimal supply chain for each country and end-use. The hydrogen required for minimum emissions is shown by the square and star symbols for the baseline and electrification scenarios, respectively. (B) The energy that would be required for hydrogen production for the baseline and electrification minimum emissions scenarios. (C) The calculated emissions abatement potential and demand for each sector for the baseline and electrification scenarios based on the minimum emission pathway.

However, if electrification is prioritised, greater emissions reductions can be achieved using less hydrogen. Assuming renewable electricity to have an emissions intensity of 15 g CO_2_e/kWh, it is found that a combined hydrogen and electrification scenario could reduce emissions by 280 Mt CO_2_e/a in the optimal scenario, with demand for hydrogen 6.0 Mt CO_2_e/a (★). This would reduce the energy required for hydrogen production from over 1.6 PWh/a to 0.46 PWh/a. Discussion of the abatement potential of nonoptimal supply chain configurations can be found in section 3.5 of the SI.

### Prioritization of Renewable Energy Use

In Figure 4 the uses are prioritised based on the reduction of emissions per unit of hydrogen. However, this does not show how renewable energy use should be prioritised. Figure 5 shows that renewable energy should be prioritised for most direct electrification applications before hydrogen production as greater emissions reductions are possible per unit of energy. Use of hydrogen for primary steel otherwise would mitigate the most emissions per unit of renewable energy versus current emissions. The results of emissions abatement potential compared to the electrification are presented in section 3.6. of the SI.

**Figure 5:**
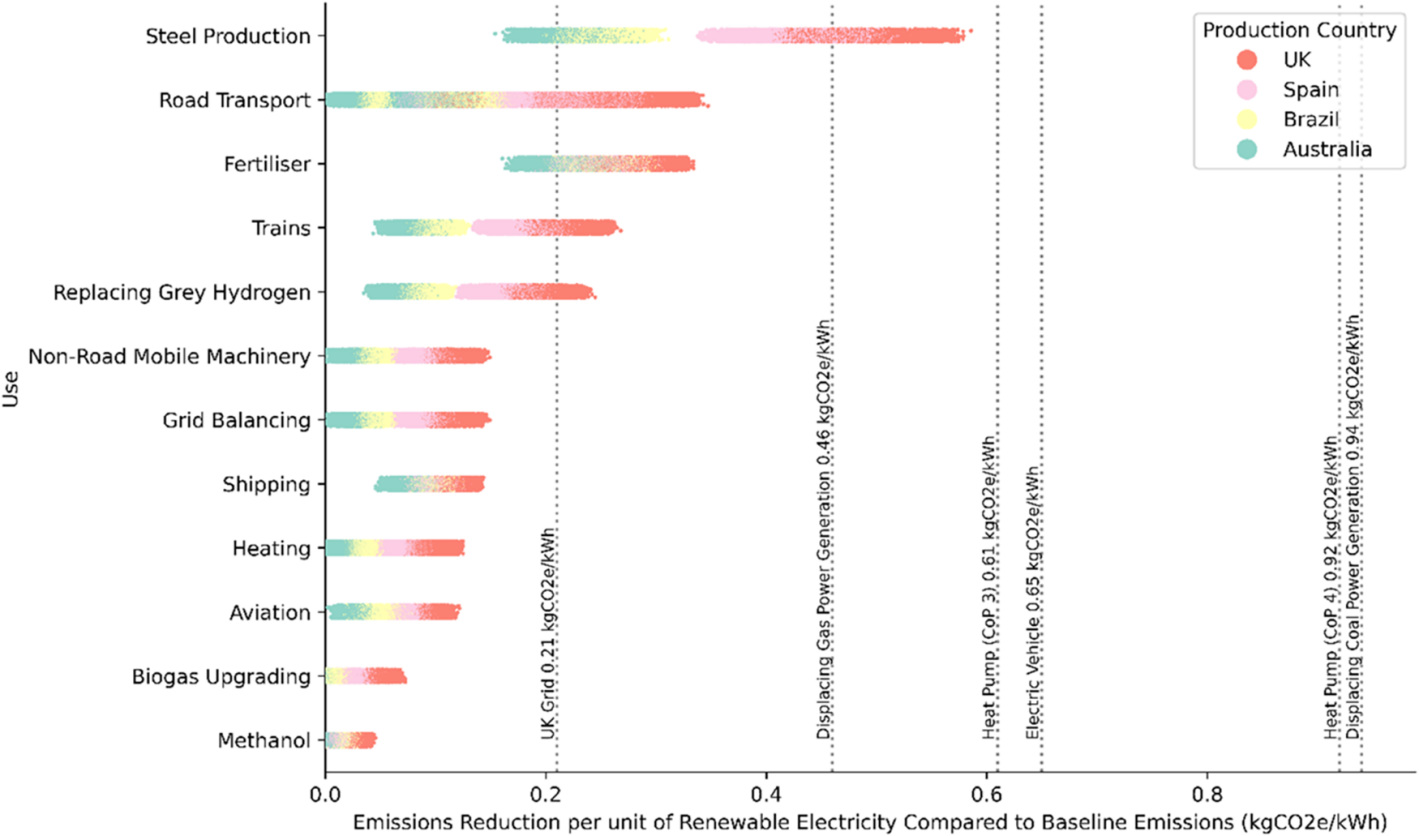
Emissions reduction potential of renewable electricity. Vertical lines refer to different applications of direct electrification to show the trade-off between using electricity directly and using electricity to produce hydrogen. Each point represents the emissions reduction potential of hydrogen use, depending on the country of hydrogen production and supply chain. Reduction per unit of electricity compares the hydrogen/electrification emissions intensity to the average baseline emissions intensity of the application stated.

## Discussion

The results show that hydrogen can reduce emissions from all the sectors considered if hydrogen that meets the UK Low Carbon Hydrogen Standard (2.4 kg CO_2_e/kg H2) can be supplied. However, for all applications which can be electrified, direct use of renewable power would lead to greater emissions savings. This is true even if hydrogen is produced using renewable electricity in the UK, the lowest emission pathway. Therefore, hydrogen use should be prioritised for applications which cannot be electrified including fertilizer production, aviation, hydrogenation, shipping, and methanol production.

Optimising the application of hydrogen and electrification could reduce emissions by up to 280 Mt CO_2_e/a based on 100 yr GWP. This is a 95% reduction from current emissions for the sectors considered. However, this scenario would require 6.0 Mt H_2_/a, which is seven times the current production of fossil hydrogen in the UK. Given the current deployment rates of nonemitting electricity in the UK, it is unclear whether enough power supply will be available to produce this amount of green hydrogen domestically and enable extensive electrification. If the analysis was conducted using shorter time horizon, e.g., 20 yr GWP, the impacts of hydrogen supply would increase, potentially decreasing the emissions reduction.

To alleviate potential capacity constraints, and enable economic hydrogen production, both the UK Hydrogen Strategy and IEA Net Zero by 2050 report suggest that increased global trade of hydrogen is likely in the long term.^1,4^ However, the results shown here suggest that most imported hydrogen supply chains are likely to exceed the UK Low Carbon Hydrogen Standard. This agrees with the results of past work where offshore transmission was found to significantly increase the emissions intensity of hydrogen supply chains.^5^ In many instances, it is found that importing hydrogen to the UK could lead to higher emissions than current production. However, regions such as South America and North Africa are likely to gain economic advantage through hydrogen exports due to low production costs^35^ so these options may still be pursued. In these instances, the carriers enabling lowest emissions are found to be ammonia or liquified hydrogen depending on the export location. Increased imports of ammonia are particularly likely as there are multiple direct uses which would avoid the need for conversion back to hydrogen, reducing the impact of transmission on the overall emissions intensity.

The hydrogen use cases that could enable the largest emissions reductions compared to current processes were found to be steel and fertilizer production, followed by jet aviation and road transport. Methanol production and biogas upgrading, followed by heating, were found to have the lowest emissions abatement. If electrification is prioritized, then use of hydrogen for road transport and steel production is no longer optimal in terms of energy efficiency or emissions intensity. However, other barriers to electrification, such as the availability of secondary steelmay result in some hydrogen use but were not considered in this study.

It is also unlikely that, in the short term, domestic supply can be prioritised to fertilizer production and jet aviation. Large scale ammonia production ceased in 2023 in the UK, as it was suggested to be more economic to import low carbon ammonia,^36^ and use of hydrogen for LH_2_ powered jet aviation is not yet technologically feasible.^37^ If direct air capture (DAC) becomes commercially viable, hydrogen could be used to produce synthetic aviation fuels resulting in lower emissions.^38^ However, DAC has not yet been demonstrated at scale.^39^ Therefore, initial use of low emission hydrogen in the UK should be prioritised for replacing gray hydrogen where electrification is not an option, with focus on overcoming barriers to deployment for other high potential sectors such as production of synthetic aviation fuel.^3^

This work required the use of assumptions on a vast number of system variables as detailed in section 2 of the SI. As the accuracy of these assumptions is an inevitable limitation, a sensitivity analysis was conducted to assess the impact on the emissions intensity of supply chains, to changes to the input variables. However, there are other sources of uncertainty which were not addressed, such as the future demand for products and services due to societal changes. Additionally, alternative decarbonization pathways other than electrification were not considered, but they could further reduce the role of hydrogen.

Further limitations existed when calculating the emissions abatement potential. For example, the current emissions were calculated based on the average energy intensities for each sector to reduce the complexity even though within sectors such as road transport there is a wide range of energy use profiles for each vehicle type. It was also assumed that either the whole sector could be electrified (e.g., steel production), or none (e.g., high temperature heat).

Further work is needed to increase the granularity of the analysis for each sector, in enough detail to find the degree of electrification possible based on each subsector and process. Priority should be given to boundary applications where hydrogen use, or electrification are found to have similar emissions abatement potential to inform policy decisions. Additionally, the economic feasibility of different hydrogen and electrification pathways should be compared since this may enable the identification of a cost-optimal solution.

## Supplementary Material



## Notes

Further details of the method and scope of analysis. Along with results of the emissions intensity of ammonia and methanol supply chains depending on transmission vector, maximum emissions abatement potential based on electrification with grid emissions, range of emissions abatement potentials from nonoptimal supply chains, Sobol Indices and emissions abatement per unit of renewable energy (PDF)
