# Supplementary material for: The Potential Role of Hydrogen in Decarbonization: Exploring Global Supply Chain Impacts and Hydrogen Use in the United Kingdom
Source: Environ Sci Technol. 2025 Jul 15;59(29):15070–9. doi: 10.1021/acs.est.4c14368 (PMC12312176; doi:10.1021/acs.est.4c14368)
Supplement: Supplementary file 1 [file es4c14368_si_001.pdf]

The potential role of hydrogen in decarbonisation: exploring global supply chain impacts and hydrogen use in the United Kingdom - Supplementary Information

Alice Bennett*[a], André Cabrera Serrenho[a]

a. Department of Engineering, University of Cambridge, Trumpington Street, Cambridge, CB2 1PZ, United Kingdom

Corresponding author:

**ab2472@cam.ac.uk*

Summary: 68 pages, 11 figures, 58 tables

Document Description

The supplementary information to the paper "The potential role of hydrogen in decarbonisation: exploring global supply chain impacts and prioritising hydrogen use in the United Kingdom" contains two components. The first component is this document which contains further details of the methods, assumptions and results related to the paper. The second component is a github repository (https://github.com/ab2472/GlobalH2supply), which contains the Python files used to create the results along with the raw data.

Contents

1.Method 4
1.1. Supply Chain Definition 4
1.2. Emissions Intensity Calculations 6
1.3. Uncertainty Analysis 6
2. Scope 8
2.1. Locations 8
2.2. Energy Inputs 10
2.3. Utilisation rates associated with use of renewable energy sources 14
2.4. Production Methods 15
2.5. Conversions 19
2.6. Transmission Methods 23
2.7. Storage 34
2.8. Uses 39
3. Results 54
3.1. Emissions Intensity of Methanol Supply Chains 54
3.2. Emissions Intensity of Ammonia Supply Chains 55
3.3. Impact of Decarbonised Transport 56
3.4. Sobol Analysis 57
3.5. Emissions abatement potential versus current grid emissions 58
3.6. Maximum range of emissions abatement potential 59
3.7. Emissions abatement potential per unit of renewable energy 60
References 61

Figures

Figure 1 Model Process Flow 4
Figure 2 Supply Chain Stages 5
Figure 3 Supply Chain Algorithm 6
Figure 4 Energy Source Assignment Process 6
Figure 5 Emissions intensity of both domestic and imported methanol supply chains for the lowest emission supply chain 54
Figure 6 Emissions intensity of both domestic and imported ammonia supply chains for the lowest emissions supply chain based on the production country, main transmission vector, and production method. 55
Figure 7 Emissions intensity of both domestic and imported hydrogen supply chains with decarbonised transport 56
Figure 8 Sobol Indices 57
Figure 9 A.Decarbonisation potential of hydrogen use 58
Figure 10 A. The range of total emissions depending on hydrogen supply based on use of ATR with CCS to produce hydrogen 59
Figure 11 A. The emissions reduction potential of renewable energy used to produce hydrogen compared to electrification. 60

1. Method

To systematically compare all possible supply chains given the scope descried in section 2 the model described by Bennett et al. [1] has been extended to include international production pathways and the end use of hydrogen. The full python code is available on github at: https://github.com/ab2472/GlobalH2supply. Within the text of the paper the key parts of the method are described with more detail of the supply chain definition, emissions intensity calculations and uncertainty analysis provided here. In figure 1 a diagram displaying the model process is shown. At step 1 data was collected for each of the technology options chosen within the scope of the case study from a range of sources included literature, technical reports and industry data. This was then inputted to the model, and the possible supply chains created using the algorithm described in section 1.1. of the SI. The losses, energy demands and emissions intensity (based on 100yr GWP of GHGs) were calculated for the supply chains up to the end use boundary as described in the methods section of the main paper. The end use impacts were then calculated to allow the emissions abatement potential of hydrogen use in the UK to be estimated.

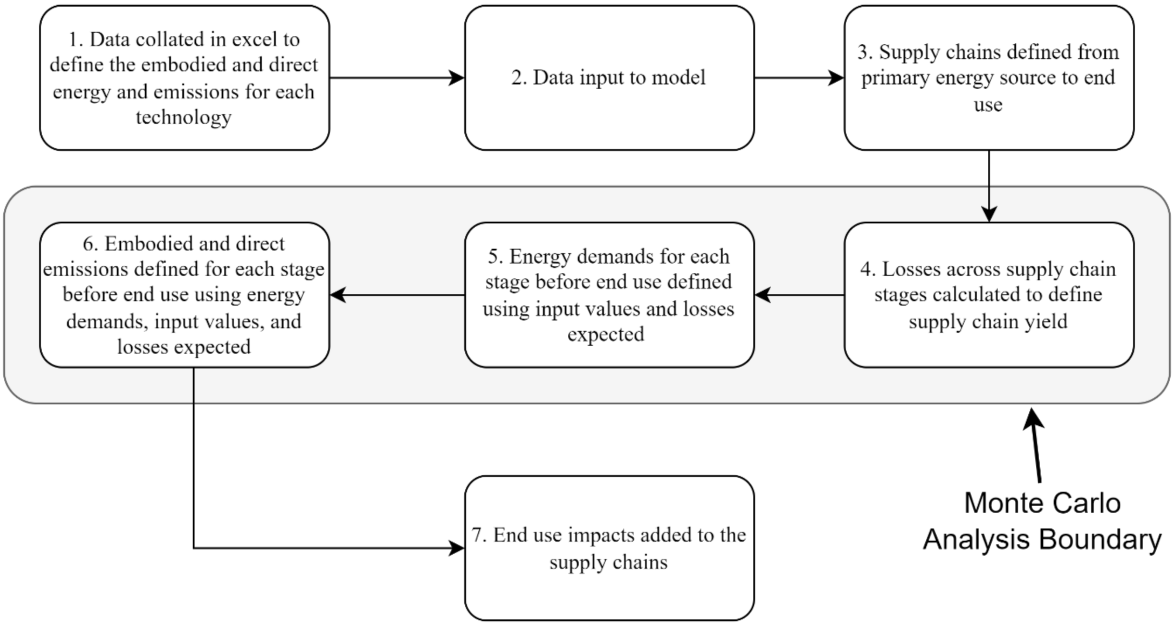

Figure 1 Model Process Flow

1.1. Supply Chain Definition

In Figure 2 the supply chain stages are shown for domestic and international production. The conversion stages may not all be required, depending on the pathway as shown by the dashed boxes and lines. The supply chains were defined based on an algorithm described below and summarised in figure 3.

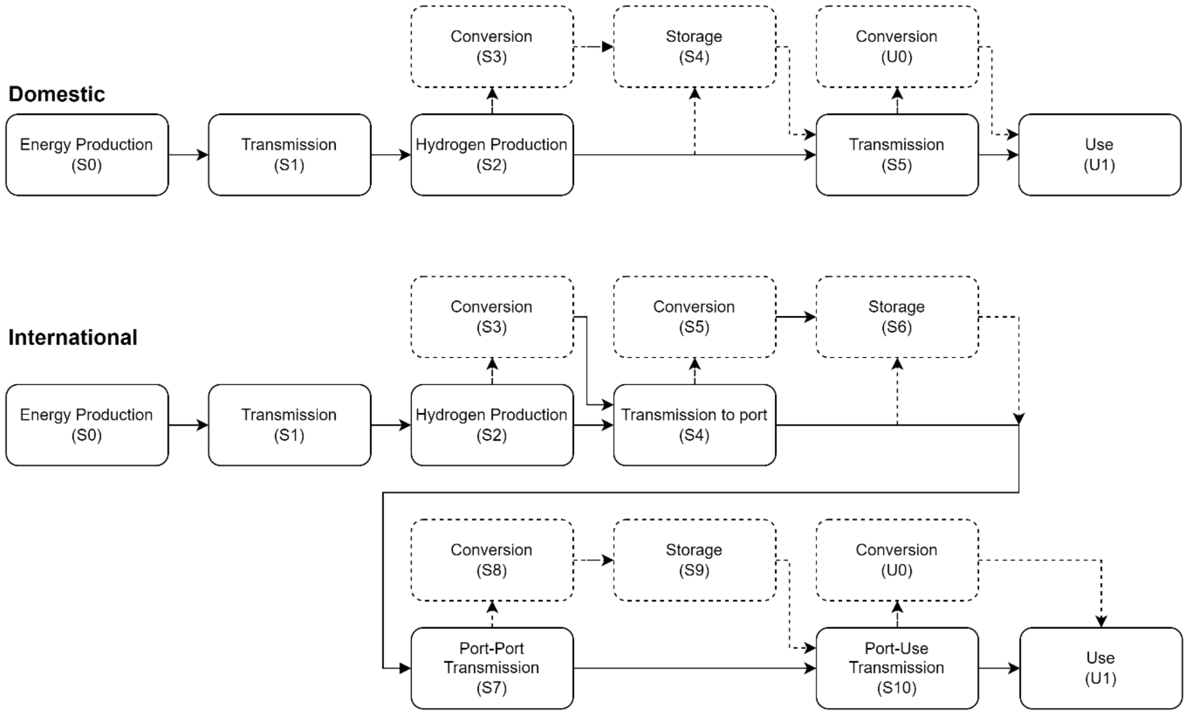

Figure 2 Supply Chain Stages

To create the supply chains firstly, the possible transmission pathways were defined:

1. Locations in the same country, and of concurrent stages, are linked to form onshore transmission paths
2. Locations in different countries, but with "Offshore" tags (e.g. ports) and linked to locations in the UK with "Offshore" tags are linked to form offshore transmission paths

These are then combined to form supply chains, with five stages for international supply chains and three stages for domestic supply chains as described below:

1. Primary energy generation to hydrogen production (both)
2. Hydrogen production to port (international only)
3. Port to port (international only)
4. Port to end use (international only)
5. Hydrogen production to end use (domestic only)

The supply chains were limited to only include at most one of ammonia or methanol, due to the energy required and losses from cracking making it very unlikely a supply chain including both could be optimal [2].

The technology option for each transmission stage could then be defined based on the following factors:

1. Distance
2. Vector (LH2, H2, Electricity, Natural Gas, Methanol, Ammonia)
3. Location (Onshore or Offshore)

In some cases, more than one transmission option was possible for each transmission pathway, with all viable possibilities were considered within the results. The production method and energy sources were then added to the supply chains by comparing the available energy sources at the location to those defined in the inputs and the energy type to that required for the production methods. Again, in some cases this results in multiple options for some supply chains with all the options considered.

At this stage the conversions required between stages were also defined if different carriers were used such as ammonia or methanol.

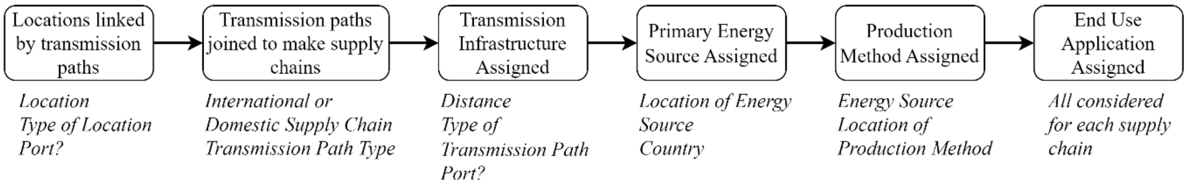

Figure 3 Supply Chain Algorithm

1.2. Emissions Intensity Calculations

Similar equations are used to calculate the emissions intensity of each stage to the energy intensity, but additionally the emissions intensity of energy use is accounted for. Before the emissions intensity can be calculated each energy use within the supply chain is assigned to an energy source. This is done using the process in Figure 4. The terms 'Base_E' and 'Base_NG' are used in the data inputs to signify unknown energy sources which are then assigned during the process to avoid assuming that the primary energy source is used as a fuel throughout the process, even where this is not viable.

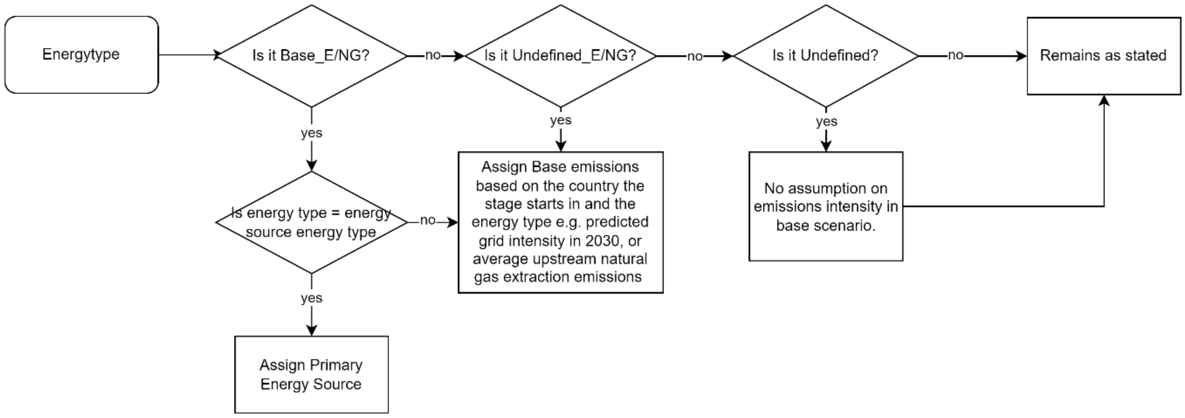

Figure 4 Energy Source Assignment Process

1.3. Uncertainty Analysis

Both Monte Carlo analysis and Sobol Analysis are used in this work to assess the impact of uncertainty. Monte Carlo sampling allows possible outcomes to be predicted given the uncertainty input values. The Sobol method allows the variation of an output, Y, due to an input, χ_i, to be

quantified. This allows us to determine which of the variables had most impact on the variance of the results of a given pathway, and which had minimal impact given the bounds defined and are therefore less important to consider. This was chosen over a local sensitivity analysis as the total effect Sobol index, S_Ti, allows the interactions between all variables to be captured. The implementation of both is as described by Bennett et al. (2024), but unlike in the previous implementation all variables included in the Monte Carlo analysis and Sobol Analysis [1]. For the Sobol Analysis the variables are grouped by the supply chain stage to decrease the uncertainty with the same computational power. Sobol analysis is described in further detail by Craglia and Cullen [25] and was implemented in the python model using the SAlib package [26], [27].

2. Scope

To allow a wide range of possible supply chains that encompasses many suggested options eight production locations, two production methods, four transmission vectors, nineteen end uses and at least three primary energy sources for each location are considered. In the following sections the rationale behind the options selected for each of the types of stage are explained in detail, and the assumptions made stated.

2.1. Locations

Three sets of location are defined in the model: the location of primary energy production, the location of hydrogen production, the location of ports for international transmission and the use location.

2.1.1. Production Locations

The production locations included within the model as the top 8 largest projects from different countries within the IEA Hydrogen Project Database [3]. These have been chosen as they represent a range of transmission distances from the UK and are geographically spread so the results can be assumed to encompass the results if other countries were selected instead.

The projects at each of the locations plan to use electrolysis to produce hydrogen, other in the case of Teesside. However, to explore the comparative impacts of electrolysis versus autothermal reforming with CCS (ATR with CCS) this will also be modelled for some of the locations with high natural gas availability (Mauritania, Kazakhstan, USA, Brazil, UK). In Table 1 the planned locations are stated.

Table 1 Production Locations [3]

Project Name	Country	Lat	Long
Western Green Energy Hub	Australia	-30.74776935	121.4650079
Megaton Moon	Mauritania	18.62110882	-12.46829637
ACME SCZONE Green Ammonia Plant	Egypt	29.69318244	32.31661541
Mauritania & BP- Nassim project	*Mauritania*	*Not included as Mauritania option already considered*	
Hyrasia one	Kazakhstan	42.79816358	53.59887962
Lacq Hydrgoen	Spain	40.17937457	-2.024929182
Teesside	UK	54.57668992	-1.252393053
Green Energy Park	Brazil	-7.682173767	-42.52223697
Hydrogen City Phase 2	USA	27.67118901	-98.61402482

2.1.2. Ports

Ports are required for large scale transmission of hydrogen (or hydrogen carriers) globally. The ports have been chosen based on the proximity to the hydrogen production locations. It has been assumed in all cases that existing ports will be utilised, though it is possible that dedicated ports could be constructed it would increase the capital infrastructure required for hydrogen transmission. The port names and locations are listed in Table 2.

Table 2 Port Locations

**Country**	**Name**	**Lat**	**Long**
USA	Corpus Christi, Texas	27.78814	-97.4078
France	Merville-Franceville-Plage	49.27859	-0.24764
Russia	Black Sea	44.74322	37.76719
Egypt	Ataka	29.643411	32.350849
Brazil	Port of Pecem	-3.546179	-38.8352
Australia	Port Bonython	-32.98733	137.7566
Mauritania	Port Autonome de Nouakchott	17.99245	-16.0306
UK	Grain LNG Port	51.43294	0.701094
UK	Teesside Port	54.63909	-1.14058

2.1.3. Primary Energy Source Locations

The locations of primary energy source production have been decided based either on existing energy source locations, or if it was stated in the IEA Hydrogen Project Database [3] that new generation would be part of the project then it has been assumed to be closely located to production to reduce transmission distance. The locations and energy types available at each are listed in Table 3.

Table 3 Energy Source Locations

Country	Location	Energy Type	**Lat**	**Long**
UK	North Sea, near Shetland Islands	Offshore Wind (UK)	59.825577,	-0.986521
UK	Location of Offshore NG installation North Sea	NG (UK), LNG	58.45053	-1.85808
UK	Open Land near Teesside, proxy for direct connection to onshore wind farm with minimal transmission	Onshore Wind (UK)	54.60179	-1.3851
Spain	Planned offshore wind project near hydrogen production.	Wind (SP)	43.591314	-5.959623
Spain	Open Land near hydrogen project	Solar (SP)	43.52689	-5.93675
Kazakhstan	Location of planned hydrogen project	Wind (KA), Solar (KA), NG (KA)	43.59176	52.71053
Brazil	O Parqu Solar Nova Olinda	Solar (BR), NG (BR)	-8.204538	-42.554876
Brazil	Lagoa dos Ventos	Wind (BR)	-8.925811	-41.609043
Australia	Location of planned projects	Solar (AU), Wind (AU)	-30.2601	127.6859
Mauritania	Co-located with hydrogen project	Solar (MA), Wind (MA), NG (MA)	18.62110882	-12.46829637
USA	Permian Basin Texas	NG (USA)	32.414829	-102.965840
USA	Solar Farm Location	Solar (USA)	29.269278	-98.456278
USA	Wind Farm Location	Wind (USA)	27.481247	-98.971620
Egypt	Co-located with hydrogen project	Solar (EG), Wind (EG), NG (EG)	29.69318244	32.31661541

2.1.4. Use Locations

One use location is considered at Teesside.

2.2. Energy Inputs

For each country three primary energy sources are considered: solar, wind and natural gas. The current grid intensity and 2030 grid intensities are also included as inputs to the model for cases where it is decided that it is unreasonable to assume that any of the primary energy sources could be utilised, the emissions factors for each country are detailed in section 2.2.1. The emissions intensities of other fuels such as diesel and marine fuel oil are also inputted into the model for use in some of the pathway stages where specified. These are detailed in section 2.2.2.

Finally, as in this work it is assumed that electrolysers are not grid connected, and no storage of primary energy is assumed, the utilisation rates of the electrolyser depending on the type of renewable energy and location are important to consider. The implementation of this is explained further in section 2.2.3.

2.2.1. Primary Energy Sources – Emissions Factor

In table 4 the emissions factors for energy generated in the UK are listed, with the emissions factors for international energy production listed in table 5. Where possible the same references have been used across multiple countries so that the basis is similar across all countries where possible.

Table 4 Emissions Factors Energy Sources UK

Country	Type	Emissions Factor (gCO_2e/kWh)	Details
UK	Offshore Wind	6.4-19.5	Based on literature [4]
	Onshore Wind	6.6-13.4	Limited updated data as new construction stalled in the UK post 2015, range taken from review of other works [5]
	Solar	60-100	Range estimated from reference. [6]
	Grid (2030)	69-228	Target is a net zero power sector by 2035, assume a linear decrease between 2023 and 2035 results in an emissions intensity of 69gCO2e/kWh in 2030. [7]
	Grid Current	207	GHG Conversion Factors 2024 [8]
	NG	6-51	North Sea Transition Authority Report on Emissions Factors [9]

Table 5 Emissions Factors Energy Sources Non-UK

Country	Type	Emissions Factor (gCO_2e/kWh) base value in bold, range for MC below	Details
Australia	Onshore Wind	12.1-20.5	From reference [10]
	Solar	22-60	Range found by [6] for Oceania, no technology assumed
	Grid (2030)	479-625	Lower bound from research by Aurora Energy Research, presented by Statista [11], upper bound current emissions
	Grid (Current)	625	From research by Aurora Energy Research, presented by Statista [12]
	NG	3-10	From reference [13]
USA	Onshore Wind	10.9-23.1	From reference [10]

Country	Type	Emissions Factor (gCO2e/kWh) base value in bold, range for MC below	Details
	Solar	22-69	Range found by [6] for N. America, no technology assumed
	Grid (2030)	233-370	From research by Aurora Energy Research, presented by Statista [11]
	Grid (Current)	**370**	From reference [14]
	NG	44	From reference [15]
Spain	Onshore Wind	11.9-24.0	From reference [10]
	Solar	43-58	Range estimated from reference. [6]
	Grid (2030)	79.5-174	Lower bound from research by Aurora Energy Research, presented by Statista [16], upper bound current emissions.
	Grid Current	174	From reference [14]
Mauritania	Wind	5.3-88	From reference [10]
	Solar	47-58	Range found by [6] for Africa, no technology assumed
	Grid (2030)	361-412	Based on reduction targets across all sectors, assume lower bound that electricity generation reduces by double the overall 11% target. [17]
	Grid (Current)	464	From reference [14]
	NG	20-50, **35**	No data – field new, use global average based on IEA total emissions from Natural gas operations and total Natural Gas extraction. [18], [19]
Brazil	Onshore Wind	10.9-23.1	From reference [10]
	Solar	*29-58*	Range estimated from reference [6]
	Grid (2030)	90-96	Upper bound current emissions intensity, lower bound based on data from reference [20]
	Grid (Current)	**96**	From reference [14]
	NG	6-10	Upstream emissions only from reference [21]
Kazakhstan	Onshore Wind	18.0-42.9	Range estimated from reference [10]
	Solar	**45.2** 43.8-76.0	Range estimated from reference [6]
	Grid (2030)	400-821	Lower bound based on emission reduction commitment, upper bound current emissions [22]
	Grid (Current)	821	From reference [22]

Country	Type	Emissions Factor (gCO_2e/kWh) base value in bold, range for MC below	Details
	NG	20-50, **35**	Limited data – field new, use global average based on IEA total emissions from Natural gas operations and total Natural Gas extraction. [18], [19]
Egypt	Onshore Wind	**19.0**<br>5.3-88<br>11.7-41.6	From reference [10]
	Solar	**47.9**<br>45-56	Range estimated from reference [6]
	Grid (2030)	332-574, **400**	Lower bound based on stated commitment, upper bound current emissions [23]
	Grid (Current)	574	From reference [14]
	NG	20-50, **35**	Limited data – use global average based on IEA total emissions from Natural gas operations and total Natural Gas extraction. [18], [19]

2.2.2. Global Averages and Misc Energy Sources

- Use for auxiliary energy use assumptions such as in transmission, or where there is limited evidence to suggest other assumptions would be more valid

Type	gCO_2e/kWh	Details
Global E	400-500, **460**	IEA 2022 [24]
Global NG	170-190, **183**	GHG Conversion Factors UK [25] (Gross energy values)
Ship Fuel (Marine Fuel Oil)	250-270, **261**	
Deisel (Average Biofuel Blend)	230-250, **239**	

2.4. Utilisation rates associated with use of renewable energy sources

As stated, if electrolysis is linked to renewable energy, the utilisation rate depends on whether energy storage is available, the ratio between the capacity of the renewable energy and electrolyser, and the generation profile of the renewable energy. As in this case it is assumed that electrolysers are directly connected to renewable energy the utilisation factors should consider the type of energy and the location. This is included in the model using work by Mendler et al. [26] which calculates the optimal ratio between the electrolyser and renewable energy use for lowest levelised cost. The values used are summarised in Table 6 for each country and energy type.

Table 6 Utilisation Factors

Country	PV			Wind Onshore			Wind Offshore		
	Min	Mean	Max	Min	Mean	Max	Min	Mean	Max
Australia	0.22	0.27	0.32	0.09	0.28	0.46			
USA	0.15	0.24	0.33	0.06	0.29	0.51			
Spain	0.24	0.28	0.33	0.05	0.25	0.45			
Kazakhstan	0.28	0.30	0.33	0.03	0.29	0.55			
Brazil	0.20	0.25	0.31	0.41	0.47	0.52			
UK	0.19	0.21	0.23	0.38	0.47	0.56	0.12	0.28	0.43
Mauritania	0.30	0.31	0.32	0.40	0.48	0.56			
Egypt	0.32	0.33	0.34	0.08	0.31	0.57			

2.5. Production Methods

Two production methods are included within the model, PEM electrolysis and ATR with CCS. The assumptions used relating to both are detailed in the following sections.

2.5.1. Centralised PEM Electrolysis

- The 1MW electrolyser is predominately based on work by Bareib et al. which provides an inventory for a PEM electrolyser [27], which for the base case the 2017 values were used, while the near future values were used where used as the lower bound for MC analysis
- The 1GW electrolyser is predominately based on work by Krishnan et al. [ref]. In this case the baseline values are said to be reflectively of technology available in 2020 while the near future values may be reflective of 2030 technology.
- More updated data would have been preferable, but was not found. Material Inventories were combined with Ansys EduPack [28] and Ecoinvent data [29] to estimate the energy and emission intensity of the material use
- Balance of Plant (BOP) has been scaled by 0.9 for centralised, GW scale system, as proposed by Krishnan et al. [30]
- Onshore the impacts of maintenance have been shown to be less than 1% of lifecycle emissions for all pathways and so are not considered in this analysis [1]

Material and Process Assumptions

Table 7 PEM Electrolyser Specification

Specification	Value	Reference	MC Range	Description
BOP Lifetime (yrs)	20	[27]	20-40	Note that this will interact with the stack assumptions as the stack needs to be replaced independently of the BOP [31]
Stack Lifetime (yrs)	7	[27]	5.7-10.2	[93]
Energy Input (kWh/kg H2)	55.5	[27]	46.25-62.8	LHV, 2030, upper and lower bounds from reference [32]
H2 Losses (%)	2.05	[33]	0.1-4	[33]
Yield (%)	95	[33]	95-100	
Water Demand (kg/kg H2)	8.9	[34] Add to process energy.	8.9	
Embodied Emissions 1MW (kgCO2e)	53000	Using table 3&5	32000;53000	Using table 3&5
Embodied Energy 1MW (kWh)	242000	Using table 3&5	67000;242000	Using table 3&5
Embodied Emissions 1GW (kgCO2e)	6.6*10^8	Using table 4&5	0.75-6.6*10^8	Using table 4&5
Embodied Energy 1GW (kWh)	2.9*10^8	Using table 4&5	2.23-2.9*10^8	Using table 4&5
Maintenance emissions		Found in previous work to be insignificant for onshore production pathways.		

Table 8 Materials 1MW Stack [27], for MC analysis the bounds are 2017 and Near Future. 2017 Values are taken as the baseline.

Material (kg)	2017	Near future
Titanium	528	37
Aluminum	27	54
Stainless steel	100	40
Copper	4.5	9
Nafion®	16	2
Activated carbon	9	4.5
Iridium	0.75	0.037
Platinum	0.075	0.010

Table 9 Materials 1GW Stack [35], for MC analysis the bounds are 2020 and Near Future. 2020 values are used as the upper bound for the analysis with near future values the lower bounds.

Material (Ecoinvent)	Weight (kg/1GW)	
	2020 (kg)	Near Future (2030) (kg)
Tetrafluoroethylene {GLO}| market for | APOS, S	7979.1	2518.2
Platinum {GLO}| market for | APOS, U	167.9	7.9
Carbon black {GLO}| market for | APOS, S	251.9	11.9
Tetrafluoroethylene {GLO}| market for | APOS, S	2014.9	95.4
water, deionised {Europe without Switzerland}| market for water, deionised | APOS, U	181.9	8.6
Methanol {GLO}| market for | APOS, S	181.9	8.6
Rhodium {GLO}| market for | APOS, U	447.8	15.9
Carbon fibre reinforced plastic, injection moulded {GLO}| market for carbon fibre reinforced plastic, injection moulded | APOS, S	2758.2	1958.6
Titanium, primary {GLO}| market for | APOS, U	151320.9	0
Gold {GLO}| market for | APOS, U	86.4	0
Steel, chromium steel 18/8 {GLO}| market for | APOS, U	0	381538.5
Tantalum, powder, capacitor-grade {GLO}| market for, 2030 | APOS, U*	0	54496.4
Glass fibre reinforced plastic, polyamide, injection moulded {GLO}| market for | APOS, S	30630.1	44589.0
Steel, chromium steel 18/8 {GLO}| market for | APOS, U	250746.3	183138.5
Gold {GLO}| market for | APOS, U	242.0	0
Tantalum, powder, capacitor-grade {GLO}| market for, 2030 | APOS, U*	0	156949.7
Aluminium alloy, metal matrix composite {GLO}| market for | APOS, S	19047.7	6731.6

Table 10 Materials BOP (Container for 1MW Stack) [ref krishnan] [27], the 2020 values are used as upper bounds while the near future values are used as lower bounds for the analysis.

**Materials**	**Decentralised (t/MW)**		**Centralised (t/MW)**	
	**2020 (t)**	**Near Future (t)**	**2020 (t)**	**Near Future (t)**
Low alloyed steel	4.8	2.4	4.32	2.16
High alloyed steel	1.9	0.85	1.71	0.765
Aluminum	< 0.1	0.05	0.09	0.045
Copper	< 0.1	0.05	0.09	0.045
Plastic	0.3	0.15	0.27	0.135
Electronic material (power, control)	1.1	0.55	0.99	0.495
Process material (adsorbent, lubricant)	0.2	0.1	0.18	0.09
Concrete	5.6	2.8	5.04	2.52

2.5.2. ATR with High CCS

- Process assumptions taken mostly from Oni et al. [36]
- Materials (and embodied emissions) are assumed to be similar to SMR which is described by [37], in previous research they have been shown to be less than 3% of the lifecycle emissions overall so higher confidence in data is unlikely to alter the key results
- Energy required to store CO2 Is included, but losses and compression along a CO2 pipeline (and the CO2 pipeline itself) are not accounted for
- Similarly, to electrolysis, O&M has been shown to contribute a negligible amount to the overall lifecycle emissions of hydrogen production via ATR so it is not included
- Carbon capture boundary includes compression and injection energy requirements only, assuming injection into a nearby depleted gas field. Transportation would also be required, which will increase the energy requirements and losses so the CCS assumptions in this work are representative of a minimum bound, given a 90% capture rate, with actual energy use and emissions intensity likely to be increased.

Table 11 Process Specification ATR with CCS

Specification	Value	Reference	MC Range	Description
Lifetime (yrs)	20	[37]	20-50	Upper bound EcoInvent chemical factory [38]
Electricity (kWh/kg H2)	3.59	[36]	3.6-4.07	Base value lower bound, Upper Bound [39]
NG (kWh/kgH2)	41.7	[36]	41.7-49	Base value lower bound, Upper Bound [39]
H2 Losses (%)	0.55	[33]	0.1-1	[33]
Yield (%)	97	[37]	94-100	
Capacity (t/day)	607	[37]		Not included in MC, changes in load factor accounted for separately.
Direct Emissions (kgCO2e/kgH2)	0.62	[36]	0.45-0.7	Low from [40], high from [41] (Direct emissions only)
Load Factor (%)	90	[37]	80-100	No Reference
Water (kg/kg)	20.93	[39]		Not included in MC
Capture Rate (%)	90	[36]		Not directly inputted into model (direct emissions inputted)

Table 12 Carbon Capture Inputs

Process	Value	Reference	MC Range	Description
Injection Energy Requirement (kWh/tCO2)	24	[42]		Not included in MC
Compression to 110bar from 1bar (kWh/tCO2)	114	[42]		Not included in MC
Captured CO2e (kgCO2e/kgH2)	7.8	[36]		

Table 13 Material Inventory - assumed to be the same as SMR plant from [37], no data for lower bound, so use same ratio used as difference between current and near future masses for electrolyser BOP

Materials	t	Future
Concrete	10242	5100
Steel	3272	1636
Aluminium	27	13
Iron	40	20

2.6. Conversions

For transmission, storage and use hydrogen is converted to other chemical compounds such as ammonia and methanol, or liquified hydrogen. In the model, it is assumed that hydrogen can be converted to or from compressed hydrogen to liquified hydrogen and back to any one of the compounds. If a pathway requires conversion from one compound to another, hydrogen is assumed to be a mid-stage in the process, so the energy and emissions impacts are assumed to be additional. All values are inputted to the model per kg hydrogen equivalent, using the hydrogen content by weight in the carrier as the conversion factor.

2.6.1. Liquefaction

Table 14 Process Specification Liquefaction

Process	Value	Reference	MC Range	Description
Energy (liquefaction) (kWh/kgH2)	13.5	[43]	12-15	[43]
Losses (liquefaction)	0.5	Same as emissions rates [33]	0.14-1.65	Same as emission rates
H2 emissions	0.34	[33]	0.14-1.65	[33], [44]
Losses regasification	0.5	[44]	0.14-1.65	Same as for liquefaction
Energy (regasification) (kWh/kgH2)	0	[44]	0-0.6	[44]

Table 15 Materials Liquefaction Plant [45]

Process	Value	Reference	MC Range	Description
Capacity Factor (%)	80		60-100	
Lifetime (years)	30	[46]	20-40	
Capacity (t/day)	50	[45]		
Steel, low alloy (t)	380			50% lower emissions assume that processes decarbonise, energy +-20%
Stainless Steel (t)	595			
Water (l/kg H2)	15			
Concrete (m3)	19029			
Aluminium (t)	140			
Copper (t)	150			

2.6.2. Ammonia

- Have assumed ammonia is used to fuel cracking – other energy could be used which would change the loss and energy input assumptions
- Limited material inventory data available, used Eco invent chemical factory construction

Table 16 Process spec Ammonia Production

**Process**	**Value**	**Reference**	**MC Range**	**Description**
Energy (synthesis) (kWh/kg NH3)	0.9	[44] pre-compression and Haber Bosch	0.9-4	Upper bound from [43]
Losses (synthesis)	5.5	[44]	1-10	[44]
H2 emissions	0.5	Compressor losses [44]	0-1	
Losses cracking	32	Part of hydrogen used as fuel for cracking [44] + compressor losses of 0.5%	30-32.9	Upper bound [33] +compression losses
Energy (cracking) (kWh/kg NH3)	1.4	[44] electricity demand for cracking and compression	1.4-2	Based on ranges for compression and cracking intensities given [47]
Embodied Energy (MJ)	2.3371e+9	Limited available data, based on Ecoinvent Chemical Factory construction [38]	1.86-2.8e9	50% lower emissions assume that processes decarbonise, energy +-20%
Embodied Emissions (kgCO2e)	1.3430e+8	Limited available data, based on Ecoinvent Chemical Factory construction [38]	0.67-1.34e9	50% lower emissions assume that processes decarbonise, energy +-20%
Lifetime	50	Limited available data, based on Ecoinvent Chemical Factory construction [38]	30-70	Limited available data
Capacity Factor	1	Limited available data, based on Ecoinvent Chemical Factory construction [38]		
Capacity (kgNH3)	25*10^8	[48]		

2.6.3. Methanol Synthesis

- Most assumptions based on work by Sollai, which splits the process into hydrogen production, CO2 capture and then methanol production [49]
- In the model hydrogen production is modelled separately, and it is assumed that CO2 is a by product input so no emissions or energy are accounted for its capture.
- Limited data for the embodied energy and emissions so Ecoinvent methanol factory construction used
- For the conversion of methanol to hydrogen available information is limited, we have assumed methanol steam reforming is used with a 90% carbon capture rate based on Kanchiralla et al. 2022 [50] and Sollai et al. 2023 [49]

Table 17 Hydrogen to Methanol Process Specification

Process	Value	Reference	MC Range	Description
Hydrogen input per kg Methanol (by weight)	0.125			
Yield (%)	59	Based on hydrogen input required and losses in system [49]	59-65	Lower bound from overall water gas shift reaction, upper bound from reference.
Energy intensity (synthesis) (kWh/kgCH3OH)	0.2	Assume CO2 is a by-product of other processes, no additional energy to capture [49]	0.2-0.9	Upper bound from [50]
Emissions (synthesis) (kgCO2e/kgCH3OH)	0	No direct emissions – assume that CO2e input required is a by product of other process.	0	
Water (kg/kgCH3OH)	0.57	[49]		
Hydrogen Emissions (%)	0	Assume all losses flared to no hydrogen emissions	0-1	Assume all losses not flared as upper bound

Table 18 Embodied Emissions (Same assumptions made for both directions of process)

Process	Value	Reference	MC Range	Description
Lifetime	30	[51]	20-50	
Capacity Factor (%)	90	[49]	0.6-1	20% range
Production Capacity (t/d)	2700	[51]		
Embodied Energy (MJ)	5.5*10^8	CED [51]	4.4-6.6*10^8	20% higher or lower
Embodied Emissions (kgCO2e)	3.8*10^7	CML 2016 v4.8 [51]	1.9-3.8*10^7	50% lower as lower bound, assumes decarbonisation will reduce emissions

Table 19 Methanol to Hydrogen

Process	Value	Reference	MC Range	Description
Hydrogen output per kg Methanol (kgH2/kgCH3OH)	0.188	[52]	0.146-0.188	20% lower, limited data found
Yield (%)	0.9	Does not account for balance in reaction, likely to be lower.	0.8-1	20% range
Energy intensity (steam reforming) (kWh/kgH2)	6.7	Steam reforming of methanol to produce hydrogen [43], co	6.7-8.04	[43]
Energy intensity – carbon capture (kWh/kgCO2)	0.96	Based on chemical absorption using amine-based solids [49]	0.83-1.39	[49]
Emissions (synthesis) (kgCO2e/kgCH3OH)	0.15	From chemical reaction, combined with 90% carbon capture [49]	0.15-0.18	20% higher, limited data found
Water input (kg/kgCH3OH)	0.56	From chemical reaction	0.56-0.68	20% higher, limited data found
Hydrogen Emissions	0	No available data found		No available data found

2.7. Transmission Methods

Transmission is needed from primary energy production to hydrogen production to end use. For import from other countries a key component is transmission offshore. The following options have been included in the scope of the case study:

- Natural gas pipeline
- Hydrogen pipeline
- Cable
- Tanker (sea transport)
    - LH2
    - NH3
    - CH3OH
- Truck (road transport)
    - LH2
    - NH3
    - CH3OH
    - CH2

2.7.1. NG Pipeline

- Embodied emissions from Ecoinvent [53]
- Limits in place for distance they can be used across, defined by looking at current max lengths of offshore and onshore pipelines

Table 20 Onshore NG Pipeline Specification

Specification	Value	Ref	MC values	Description
Capacity (million Nm3)	1.1	Ecoinvent [53]	NA	Designed Capacity
Load Factor (%)	0.7	US oil pipeline utilisation [54]	0.5-1	Low = lowest value quoted in reference, high = full utilisation [54]
Lifetime (yrs)	40	Ecoinvent [53]	40-70	Upper bound from reference [55]
Embodied Emissions (kgCO2e/km)	1.39*10^6	CML v4.8 2016 Ecoinvent [53]	0.7-1.4*10^7	Medium Confidence, if construction and production of materials decarbonised emissions will reduce significantly, bounds 50% lower.
Embodied Energy (MJ/km)	1.78*10^7	CED Ecoinvent [53]	1.44-2.16*10^7	Medium Confidence, some gain from energy efficiency possible but may be outweighed by less efficient process with lower emissions, bounds 20% higher and lower
Distance Limit (km)	5000	Based on longest onshore pipelines [56]	NA	Not applicable to uncertainty analysis.
Losses (%)	NA	Accounted for in Ecoinvent energy use	NA	NA
Energy (MJ/tkm)	1.1	Ecoinvent [57]	NA	Mature technology but limited available data on impacts of transmission as combustion results in >98% emissions intensity of NG use.

Table 21 Offshore NG Pipeline Specification

Specification	Value	Ref	MC values	Description
Capacity (million Nm3)	1.6	Ecoinvent [58]	NA	Designed Capacity
Load Factor (%)	0.7	US oil pipeline utilisation [54]	0.5-1	Low = lowest value quoted in reference, high = full utilisation [54]
Lifetime (yrs)	45	Ecoinvent [58]	40-70	Upper bound from reference [55]
Embodied Emissions (kgCO2e/km)	1.92*10^6	CML v4.8 2016 Ecoinvent [58]	0.96-1.92*10^6	Medium Confidence, if construction and production of materials decarbonised emissions will reduce significantly, bounds 50% lower.
Embodied Energy (MJ/km)	2.28x10^8	CED Ecoinvent [58]	1.82-2.73*10^8	Medium Confidence, some gain from energy efficiency possible but may be outweighed by less efficient process with lower emissions, bounds 20% higher and lower
Distance Limit (km)	1200	Based on longest offshore pipeline [59]	NA	NA
Losses (%)	NA	Accounted for in Ecoinvent energy use	NA	NA
Energy (MJ /tkm)	1.1	Ecoinvent [57]	NA	Mature technology but limited available data on impacts of transmission as combustion results in >98% emissions intensity of NG use.

2.7.2. H2 Pipeline

- Based on review of the challenges faced before reusing natural gas infrastructure hydrogen, which found that serious safety and environment risks are likely even if techno-economical barriers can be overcome, only new hydrogen infrastructure is considered [60]
- Limited Data, assume construction emissions based on Ecoinvent entries for offshore and onshore NG pipelines
- Distance limits for offshore and onshore pipelines are assumed to be the same as NG pipelines.

Table 22 Specification 1km Hydrogen Pipeline

Specification	Unit	Value	Reference	MC Values	Description
Capacity	GW	13	[61]	NA	Designed Capacity
Capacity Factor	%	0.9	[61]	0.5-1	Low = lowest value quoted in reference, high = full utilisation [54]
Lifetime	year	40	[61]	40-70	Upper bound from reference [55]
H2 losses	(%/km)	0.004	[44]	0-0.006	Lower bound = none, upper bound from cooper [33]
Drawing of pipe, steel	Kg/km	791,447.36	[61]	Min 50% lower	Same assumptions for embodied emissions ranges as NG pipelines
FBE powder coat, steel	m^2/km	3,830.23	[61]		
Galvalume® coat, pieces	m^2/km	3,660.00	[61]		
HDPE coat, extruded	kg/km	5,599.17	[61]		
Steel, low-alloyed	kg/km	791,447.36	[61]		
Energy Use Construction (Onshore)	MJ/km	3.31*10^6	[53]	2.65-3.97*10^6	Same assumptions for embodied energy ranges as NG pipelines
Energy Use Construction (Offshore)	MJ/km	2.5300e+6	[58]	2.02-3.04*10^6	
Energy use transmission	Wh/kgkm	0.426	[44]	0.142-0.426	Lower bound [61] upper bound [44]
Energy use (start and end) compression	kWh/kg	0.95	[44]	0-0.95	Lower bound no compression needed
Compressor loss	%	0.5	[44]	0-1	Lower bound no losses, upper bound double

2.7.3. Cable

- Electricity transmission, onshore or offshore

Table 23 Offshore Cable Specification

Specification	Value	Ref	MC values	Description
Capacity (MW)	600	[62][63]		
Load Factor (%)	50	[63]		
Lifetime (yrs)	40	[63]	20-50	
Embodied Emissions (kgCO2e/km)	2.64*10^5	CML v4.8 2016 [63]	50% lower as min	Same assumptions for embodied emissions ranges as NG pipelines
Embodied Energy (MJ/km)	4.68*10^6	CED [63]	20% higher and lower	Same assumptions for embodied energy ranges as NG pipelines
Distance Limit (km)	800	[64]		
Losses (%)	0.9	[65]	0.5-1.5	

Table 24 Onshore Cable Specification

Specification	Value	Ref	MC values	Description
Capacity (MW)	600	[66][67]		
Load Factor (%)	50	[67]	50-100	
Lifetime (yrs)	40	[67]	20-50	
Embodied Emissions (kgCO2e/km)	2.34*10^5	[67]	1.17*10^5	Same assumptions for embodied emissions ranges as NG pipelines
Embodied Energy (MJ/km)	3.99*10^6	[67]	3.2-4.8*10^6	Same assumptions for embodied energy ranges as NG pipelines
Distance Limit (km)	2500	link		
Losses (%)	0.9	[65]	0.5-1.5	

2.7.4. Tanker Assumptions

- Three transmission vectors considered: liquified hydrogen, ammonia, and methanol
- For all tankers two scenarios are explored. In the first scenario it is assumed they are powered by Marine Fuel. In the second it is assumed decarbonised shipping is possible and there are zero emissions from energy use. This is an overly optimistic approach but provides a lower bound on the impacts due to shipping.

Table 25 LH2 Tanker

Specification	Value	Ref	MC values	Description
Capacity (m3)	160000	[44]	160000	Specified volume, uncertainty dealt with in other variables
Capacity (kgH2)	11376000	Density 71.1kg/m3 [44]	11376000	Specified volume, uncertainty dealt with in other variables
Load Factor (%)	1	IEA	0.5-1	Minimum 50% capacity
Speed (km/hr)	29.6	16 knots [44]	20-39	Average speed 16 knots, maximum referenced as 20.5 knots so minimum ~11 knots [68]
Lifetime (yrs)	25	Based on lifetime of LNG tanker [69]	20 -37	LNG Tanker retired at 37, less likely to be retired before meets min age so skewed [70]
Embodied Emissions (kgCO2e)	3.25*10^7	Ecoinvent construction of LNG Tanker	1.63*10^7 - 3.25*10^7	If construction and production of materials decarbonised emissions will reduce significantly, bounds 50% lower.
Embodied Energy (MJ)	4.17*10^8	Ecoinvent construction of LNG Tanker	3.34-5*10^8	Some gain from energy efficiency possible but may be outweighed by less efficient process with lower emissions, bounds 20% higher and lower
Distance Limit (km)		No maximum		
Losses (%)	1.04	Loading and Unloading [44]	0.52-1.56	50% higher lower as range
Losses (%/day)	0.3	Boil off per day [44], converted to per km in model using average speed and 12 hour operation per day.	0.3-1	Min Kleinje[43 , Max Hydrogen science coalition [71]
Energy (MJ/tkm)	0.104	Kolb et al. calculated that the fuel consumption is reduced by 23% when a tanker is carrying LH2 instead of LNG. Based on Ecoinvent data for LNG. Heavy fuel oil.	0.135	Max: IEA data including capacity [72]
Energy (kWh/kgH2)	0.38	Loading and Unloading [44]	0.38-0.8	Max IEA import and export terminal energy use [72]

Table 26 Ammonia Tanker

Specification	Value	Ref	MC values	Description
Capacity (m3)	160000	[44]	160000	Specified volume, uncertainty dealt with in other variables
Capacity (kgH2)	1926176	Density 682.2 [44], 109152000 kgNH3	1926176	Specified volume, uncertainty dealt with in other variables
Load Factor (%)	1	Assume full capacity used	0.5-1	Minimum 50% capacity
Speed (km/hr)	29.6	16 knots [44]	20-39	Average speed 16 knots, maximum referenced as 20.5 knots so minimum ~11 knots [68]
Lifetime (yrs)	25	Based on lifetime of LNG tanker [69]	20 -37	LNG Tanker retired at 37, less likely to be retired before meets min age so skewed [70]
Embodied Emissions (kgCO2e)	3.25*10^7	Ecoinvent construction of LNG Tanker	1.63*10^7 - 3.25*10^7	If construction and production of materials decarbonised emissions will reduce significantly, bounds 50% lower.
Embodied Energy (MJ)	4.17*10^8	Ecoinvent construction of LNG Tanker	3.34-5*10^8	Some gain from energy efficiency possible but may be outweighed by less efficient process with lower emissions, bounds 20% higher and lower
Distance Limit (km)		No maximum		
Losses (%)	0.094	Loading and Unloading [44]	0.047-0.188	50% higher lower as range
Losses (%/day)	0.025	During Shipping [44]	0-0.025	IEA boil off rate 0 [72]
Energy (MJ/tNH3km)	0.15	Using same method as kolb, energy use increases by 12%	0.047-0.15	IEA used as bound for range [72]
Energy (kWh/kgH2)	0.028	0.08kWh/kgNH3 Unloading and loading [44]	0.02-0.028	Min IEA import and export terminal energy use [72]

Table 27 Methanol

Specification	Value	Ref	MC values	Description
Capacity (m3)	160000	[44]	160000	Specified volume, uncertainty dealt with in other variables
Capacity (kgH2)	1422000	Density at -34 degrees c 805 kg/m3 [73]. 11376000kgMeOH	1422000	Specified volume, uncertainty dealt with in other variables
Load Factor (%)	1	Assume full capacity used	0.5-1	Minimum 50% capacity
Speed (km/hr)	29.6	16 knots [44]	20-39	Average speed 16 knots, maximum referenced as 20.5 knots so minimum ~11 knots [68]
Lifetime (yrs)	25	Based on lifetime of LNG tanker [69]	20 -37	LNG Tanker retired at 37, less likely to be retired before meets min age so skewed [70]
Embodied Emissions (kgCO2e)	3.25*10^7	Ecoinvent construction of LNG Tanker	1.63*10^7 - 3.25*10^7	If construction and production of materials decarbonised emissions will reduce significantly, bounds 50% lower.
Embodied Energy (MJ)	4.17*10^8	Ecoinvent construction of LNG Tanker	3.34-5*10^8	Some gain from energy efficiency possible but may be outweighed by less efficient process with lower emissions, bounds 20% higher and lower
Distance Limit (km)		No maximum		
Losses (%)	0	Based on NH3 transport [74]	0-0.188	Upper bounds based on range for ammonia transport
Losses (%/day)	0		0	
Energy (MJ/tMeOHkm)	0.15		0.011-0.15	
Energy (kWh/kgMeOH)	0.028	Based on energy demand for ammonia loading and unloading	0.02-0.028	

2.7.5. Truck Assumptions

- Base assumptions the same for each chemical carried
- Amount carried differs depending on the density of the chemical
- Two scenarios are explored. In the first scenario it is assumed that trucks are powered by diesel. In the second it is assumed decarbonised shipping is possible and there are zero emissions from energy use. This is an overly optimistic approach but provides a lower bound on the impacts due to transport on land.

Table 28 Base assumptions - Truck

Specification	Value	Ref	MC values	Description
Load Factor (%)	100	Volumes small so assume able to use full capacity majority of the time	80-100	Lower bound 20% reduced use
Speed (km/hr)	50	IEA [72]	30-100	Speed restrictions UK
Lifetime (yrs)	12	IEA – depreciation period [72]	5-12	Other references suggest shorter lifespan [75]
Embodied Emissions (kgCO2e)	33338	lorry production, 28 metric ton, ecoinvent	16669	If construction and production of materials decarbonised emissions will reduce significantly, bounds 50% lower.
Embodied Energy (MJ)	479120	lorry production, 28 metric ton, ecoinvent	383296	Some gain from energy efficiency possible but may be outweighed by less efficient process with lower emissions, bounds 20% higher and lower
Distance Limit (km)		No limit		
Energy (kWh/tkm)	0.27	Articulated HGV (GHG conversion factors 2024) Assuming fuel is diesel.	0.2-0.27	Consumption Electric HGV, consumption FCEV [76]
Emissions (kgCO2e/tkm)	0.072	All HGVs, 100% laden (GHG conversion factors 2024)	0.04-0.072	Emissions electric HGV with renewable power [76]

Table 29 Truck (Compressed hydrogen)

Specification	Value	Ref	MC values	
Capacity (kgH2)	670	IEA [72]	670	Uncertainty accounted for in other variables
Losses (%)	0.5	Assume the same losses and energy requirements for loading and unloading as have been assumed for CH2 pipeline.	0-1	Assume the same losses and energy requirements for loading and unloading as have been assumed for CH2 pipeline.
Losses (%/day)	0.004		0-0.006	
Energy (kWh)	0.95		0-0.95	

Table 30 Truck (Liquified hydrogen)

Specification	Value	Ref	MC values	
Capacity (kgLH2)	4300	IEA [72]	4300	Uncertainty accounted for in other variables
Losses (%)	1.04	Assume the same losses and energy requirements for loading and unloading as have been assumed for shipping.	0.52-1.56	Assume the same losses and energy requirements for loading and unloading as have been assumed for shipping.
Losses (%/day)	0.3		0.2-1	
Energy (kWh)	0.038		0.38-0.8	

Table 31 Truck (Ammonia)

Specification	Value	Ref	MC values	
Capacity (kgH2)	459	IEA – 2600 kgNH3, hydrogen content 17.6% [72]	459	Uncertainty accounted for in other variables
Losses (%)	0.094	Assume the same losses and energy requirements for loading and unloading as have been assumed for shipping.	0.47-0.188	Assume the same losses and energy requirements for loading and unloading as have been assumed for shipping.
Losses (%/day)	0.025		0-0.025	
Energy (kWh)	0.028		0.02-0.028	

Table 32 Truck (Methanol)

Specification	Value	Ref	MC values	
Capacity (kgH2)	390	Compare density to Ammonia. 3100kg/MeOH. 12.5% by weight.	390	Uncertainty accounted for in other variables
Losses (%)	0	Assume the same losses and energy requirements for loading and unloading as have been assumed for shipping.	0-0.188	Assume the same losses and energy requirements for loading and unloading as have been assumed for shipping.
Losses (%/day)	0		0	
Energy (kWh)	0.028		0.02-0.028	

2.8. Storage

- Storage is likely to be required along the transmission pathways to allow high utilisation of transmission infrastructure and to ensure consistent supply given the intermittent nature of renewable energy
- Storage of primary energy is not considered within the scope of this work
- Storage of all the hydrogen vectors is considered using the base assumptions listed in Table 33based predominately on assumptions made by Kleijne et al. [44].
- Relatively optimistic values – literature suggests 50 days storage may be required to supply hydrogen at a constant hourly rate from renewable energy sources [77], IEA modelling uses assumption of 20 days storage at the import terminal [78]

Table 33 Storage duration assumptions

Stage	Value
Storage before shipping	3 days
Storage before pipeline	0.5 days
Storage before truck	3 days

2.8.1. Compressed Hydrogen Storage

- Details of storage from [79]

Table 34 Compressed Hydrogen Specifications

Specification	Value	Description	MC Values	Description
Energy intensity – Compression (kWh/kgH2)	0.71	Compression to 80 bar from 30 bar.	0-1	Lower bound no compressions needed, upper bound assumed lower efficiency of compression
Compressor Loss (%)	0.5	[44]	0-1	Lower bound, no losses, upper bound losses doubled
Losses (%/day)	0	No losses modelled during storage [79]	0-0.0001	No losses modelled

Specification	Value	Description	MC Values	Description
Energy intensity – Recompression (kWh/kgH2)	0	Included within the next transmission stage or conversions, not included here to avoid double counted.	0-0.0001	Included within the next transmission stage or conversions, not included here to avoid double counted.
Embodied Emissions (kgCO2e)	2.46kgCO2e/kg *126000	**126tonnes** of 304 stainless steel required. Emissions intensity from ICE DB (steel plate).<br><br>Energy intensity from[80]	0.75-2.46kgCO2e	EAF used for steel. Doesn't account for post processing.
Embodied Energy (GJ)	24.1GJ/t *126		440kWh/t-6700kwh/t	Energy intensity of EAF as lower bound. Doesn't account for post processing.
Lifetime (yrs)	10	[81]	5-15	50% higher or lower
Capacity (kgH2/tank)	527	[79] per use of tank, assume in constant use according to the number of days storage used.	527	No uncertainty, designed volume
Capacity Factor (%)	50	Assume in constant use, 50% full as unloaded once filled and then refills over the storage period.	25-75	50% higher or lower

2.8.2. Liquified Hydrogen Storage

- Details of storage predominately as assumed in [44]

Table 35 Compressed Hydrogen Specifications

Specification	Value	Description	MC Values	Description
Energy intensity – Compression (kWh/kgH2)	0	Conversion to liquified hydrogen accounted for in conversion stage	0	Conversion to liquified hydrogen accounted for in conversion stage
Loss in storage (%)	0.16	[44]	0-0.32	No losses, or doubled
Losses (%/day)	0.18	[44]	0.06-0.669	[82]
Energy intensity – Recompression (kWh/kgH2/day)	2.4kWh/kgH2	Reliquification of boil off gas. 0.18% per day. 1.81kwh/kgBOGH2 compression. 12kWh/kgBOGH2 reliquifaction.	1.8-2.4	Reliquification of boil off gas not done.
Embodied Emissions (kgCO2e)	2.46kgCO2e/kg *126000	Baseline the same as compressed hydrogen [79]	0.75-2.46kgCO2e/kg *126	EAF used for steel. Doesn't account for post processing.
Embodied Energy (GJ)	24.1GJ/t *126	Baseline the same as compressed hydrogen [79]	440kWh/t-6700kwh/t *126	Energy intensity of EAF as lower bound. Doesn't account for post processing.
Lifetime (yrs)	10	[81]	5-15	50% higher or lower
Capacity (kgH2/tank)	5677	Liquified hydrogen density 71.1kg/m3 [73]	5677	Range of densities 50-80kg/m3, but uncertainty accounted for in capacity factor.
Capacity Factor (%)	50	Assume in constant use, 50% full as unloaded once filled and then refills over the storage period.	25-75	50% higher or lower

2.8.3. Ammonia Storage

- Details of storage predominately as assumed in [44]

Table 36 Compressed Ammonia Specifications

Specification	Value	Description	MC Values	Description
Energy intensity – liquefaction and storage (kWh/kgNH3)	0.08	Klienje – electricity demand for liquefaction [44]	0.04;0.16	50 higher and lower
Loss in storage (%)	0.02	Klienje [44]	0-0.04	No losses, doubled losses
Losses (%/day)	0.015	Klienje [44]	0-0.03	No losses, doubled losses
Energy intensity – Recompression (kWh/kgH2/day)	0.08	Unloading energy [44]	0.04;0.16	50 higher and lower
Embodied Emissions (kgCO2e)	2.46kgCO2e/kg *126000	Baseline the same as compressed hydrogen [79]	0.75-2.46kgCO2e/kg	Baseline the same as compressed hydrogen [79]
Embodied Energy (GJ)	24.1GJ/t *126		440kWh/t-6700kwh/t	
Lifetime (yrs)	10	[81]	5-15	50% higher or lower
Capacity (kgH2/tank)	54450	Density at -34 degrees c 682.8kg/m3. Hydrogen density 6.6kg/m3		
Capacity Factor (%)	50	Assume in constant use, 50% full as unloaded once filled and then refills over the storage period.	25-75	50% higher or lower

2.8.4. Methanol Storage

- Details of storage predominately as assumed in [44]

Table 37 Methanol Specifications

Specification	Value	Description	MC Values	Description
Energy intensity – Compression (kWh/kgH$_2$)	0	No energy consumption shown in literature between production and storage	0	No energy consumption shown in literature between production and storage
Loss in storage (%)	0	No losses modelled along pathway [73]	0	No losses modelled along pathway [73]
Losses (%/day)	0	No losses modelled along pathway [73]	0	No losses modelled along pathway [73]
Energy intensity – Recompression (kWh/kgH$_2$/day)	0.00627	Discharging energy to onward path due to pump use [73]	0-0.012	Doubled, or none
Embodied Emissions (kgCO$_2$e)	2.46kgCO$_2$e/kg *126000	Baseline the same as compressed hydrogen [79]	0.75-2.46kgCO$_2$e/kg	Baseline the same as compressed hydrogen
Embodied Energy (GJ)	24.1GJ/t *126		440kWh/t-6700kwh/t	
Lifetime (yrs)	10	[81]	5-15	50% higher or lower
Capacity (kgH$_2$/tank)	64300	Density at -34 degrees c 805 kg/m3. Hydrogen density 6.6kg/m3[73]		
Capacity Factor (%)	50	Assume in constant use, 50% full as unloaded once filled and then refills over the storage period.	25-75	50% higher or lower

2.9. Use

Hydrogen can be used to produce heat through combustion, electricity vis electrolysis, or used as a feedstock for chemical processes such as ammonia production. The possible uses of hydrogen have been summarised in the hydrogen ladder by Liebreich [83]. Using a similar methodology to Ghadim et al. [84] the total demand, current impact and hydrogen required for each end-use type have been summarised below. The results presented are based on the current demand in each sector and do not account for changes in usage patterns. However, in cases where electrification is likely to be viable as a decarbonisation pathway the impact of hydrogen use is compared to both the current process and an electrified process.

The assumptions use the 2024 GHG conversion factors as one of the key data sources, as well as the DUKES Energy statistics.

2.9.1. Scope

All rungs of the hydrogen ladder are listed in the table along with whether they have been included or not in this assessment.

Table 38 End Uses Scope

Rung	Use	Included in Model	Details
A	Fertiliser	Y	Estimate using Ammonium Nitrate production specifications (most common in the UK [85])
	Hydrogenation	Combined into one category.	Emissions basis based on replacing current grey hydrogen production and use in the UK. Assumed that use for other purposes is minimal as no domestic ammonia production.
	Hydrocracking		
	Desulphurisation		
	Methanol	Y	
B	Shipping	Y	Include as one option only, as other sector is double counted. Ammonia chosen as expected to have the largest contribution according to estimates from the IEA.
	Jet Aviation	Y	Included based on direct use of liquified hydrogen, other options such as syngas are possible.
	Chemical feedstock	Combined with other category	Included in replacing current hydrogen production category.
	Iron and Steel	Y	
	Long duration grid balancing	Y	Storage in salt caverns the chosen technology.
C	Coastal and river vessels	Combined with other category	Included in local ferries
	Non-Road Mobile Machinery	Y	UK statistics on non-road mobile machinery include military aviation and shipping, this category intended for tractors, construction vehicles so base energy demand for the sector is based on red diesel consumption prior to ban instead.

Rung	Use	Included in Model	Details
	Vintage and Muscle Cars	N	Very small proportion of energy use and emissions in the UK – less than 0.05% [86], assumed similar potential to other vehicles.
	Biogas Upgrading	Y	
D	long distance trucks and coaches	Y	HGVs only – coaches assumed to be included within bus assumptions.
	High Temperature Heat	Y	
	Generators	N	If assume generator use is all auto generator electricity production then less than 0.005% of emissions int the UK.
E	Regional Trucks	Combined with other category	Included in long distance trucks
	Commercial Heating	Y	
F	Island Grids	N	Locations in the UK with island grids will be able to transition to predominately renewable electricity due to renewable potential.
	Short Duration Grid Balancing	Combined with other category	included in long duration balancing
	Light Aviation	Combined with other category	included in aviation
	Remote and Rural Trains	Y	All diesel trains will be switched hydrogen
	Local Ferries	Combined with other category	Combined into shipping as cannot separate the energy use between use for ferries and freight.
	Light Trucks	Y	
	Bulk Power Imports	N	Intend to continue importing electricity (and exporting) in energy strategy, use of hydrogen accounted for under long duration energy balancing.
	UPS (Uninterruptable power supply)	N	No available data on quantity installed, assumed to be negligible based on energy not supplied [87]
G	Metro Trains and Buses	Y	Buses and coaches only.
	Urban Delivery and Taxis	Combined with other category	Included in cars
	2 and 3 wheelers	Y	
	Cars	Y	
	Bulk E fuels	N	Limited use cases beyond already covered in industrial cases covered by other topics
	Mid/Low Temperature Heat	Y	

Rung	Use	Included in Model	Details
	domestic heating	Y	
	Power Generation using non-stored hydrogen	N	Converting electricity to hydrogen to electricity for immediate use will not occur.

2.9.2. Data Assumptions for the end uses

Table 39 Fertiliser Use - Nitrogen

Input	Value	Description
Annual Demand (Mt Nitrogen)	0.862	Based on demand for nitrogen fertiliser. Though this could be split between different chemical compounds such as urea, in this work baseline assumption that all supplied as ammonium nitrate.<br><br>https://www.fao.org/faostat/en/#data/RFN/visualize
Baseline Emissions (kgCO2e/kg AN)	1.5	Ammonium nitrate production in Europe (Ecoinvent) https://ecoquery.ecoinvent.org/3.9.1/cutoff/dataset/3495/impact_assessment
Ammonia demand (kgNH3/kgAN))	0.203	https://ecoquery.ecoinvent.org/3.9.1/cutoff/dataset/3495/exchanges
Other inputs	Electricity 0.08kWh/kgAN<br>Other Energy: 1.4MJ<br>Nitric Acid: 1.0*0.75	https://ecoquery.ecoinvent.org/3.9.1/cutoff/dataset/3495/exchanges<br><br>Based on difference between ammonia production and AN production.
Min Viable Unit	0	Assume no minimum as is replacing a current use of hydrogen

Table 40 Fertiliser Production - Phosphate

Input	Value	Description
Annual Demand (Mt Phosphate)	0.11	Based on demand for phosphate fertiliser. Though this could be split between different chemical compounds but in this work it is assumed to be supplied from diammonium phosphate. (DA – 46% content phosphate by mass)<br><br>https://www.fao.org/faostat/en/#data/RFN/visualize
Baseline Emissions (kgCO2e/kg DA)	1.48	diammonium phosphate production in Europe (Ecoinvent) https://ecoquery.ecoinvent.org/3.9.1/cutoff/dataset/22783/impact_assessment
Ammonia demand (kgNH3/kgkg DA)	0.22	https://ecoquery.ecoinvent.org/3.9.1/cutoff/dataset/3495/exchanges
Other inputs	Electricity 0.05kWh/kgkgDA<br>Other Energy: 0.4MJ<br>Phosphoric Acid: 0.85 kg (0.84kgCO2e/kg)	https://ecoquery.ecoinvent.org/3.9.1/cutoff/dataset/3495/exchanges<br><br>Based on difference between ammonia production and DA production.
Min Viable Unit	0	Assume no minimum as is replacing a current use of hydrogen

Table 41 Current Hydrogen Use – Refinery and Other chemicals

Input	Value	Description
Annual Demand (Mt H2)	0.195	https://observatory.clean-hydrogen.europa.eu/sites/default/files/2023-05/Chapter-2-Hydrogen-Supply-and-Demand-2021.pdf
Baseline Emissions (kgCO2e/kgH2)	12.5	IEA global average grey hydrogen
Hydrogen Demand (kgH2/kgH2)	1	Direct replacement of hydrogen
Other Inputs	NA	Direct replacement of hydrogen
Min Viable unit (kg H2)	0	Assume no minimum as it is replacing a current use.

Table 42 Methanol Production

Input	Value	Description
Annual Demand (kt CH3OH)	32	Sales 2023 – exports exceed imports based on Trade Data so not included. https://www.ons.gov.uk/businessindustryandtrade/manufacturingandproductionindustry/datasets/ukmanufacturerssalesbyproductprodcom (From https://observatory.clean-hydrogen.europa.eu/sites/default/files/2023-05/Chapter-2-Hydrogen-Supply-and-Demand-2021.pdf not apparent there is any demand in the UK)
Baseline Emissions (kgCO2e/kgcH3OH)	**0.944**	https://pubs.rsc.org/en/content/articlehtml/2024/ya/d4ya00316k
Hydrogen Demand (kgH2/kgH2)	**0.208**	https://pubs.rsc.org/en/content/articlelanding/2024/ya/d4ya00316k#:~:text=This%20LCA%20scrutinizes%20the%20environmental,significant%20potential%20for%20carbon%20neutrality.
Other Inputs		Accounted for in conversion to CH3OH
Min Viable unit (kg H2)	**0**	No minimum, replacing current use

Table 43 Shipping - Ammonia

Input	Value	Description
Annual Demand	2.79 Mt HFO	Fuel use = 2Mt MGO, 0.7Mt MFO env0101, converted all to HFO equivalent based on thermal energy content.
Baseline Emissions	3.25 kgCO2e/kg HFO	GHG conversion factors, general cargo average
Hydrogen Demand	2.07 kgNH3/kg HFO	https://iopscience.iop.org/article/10.1088/2634-4505/ad097a/pdf
Other Inputs	NA	
Min Viable unit (kg H2)	51kt	Targeted 5% by 2030, take this to be minimum supply viable https://www.imo.org/en/MediaCentre/HotTopics/Pages/Cutting-GHG-emissions.aspx

Table 44 Jet Aviation

Input	Value	Description
Annual Demand	11.1Mt	Env0101 Aviation turbine fuel
Baseline Emissions (kgCO2e/kg)	3.18kgCO2e/kg	GHG Conversion Statistics
Hydrogen Demand (kgH2/kgH2)	0.36kgH2/kg	Thermal basis – LH2 to Aviation fuel
Other Inputs	NA	
Min Viable unit (kg H2)	1Mt	Target 2.6% fuel from renewable hydrogen by 2030 - not necessarily direct hydrogen use https://www.cleanenergyministerial.org/content/uploads/2022/07/aspirational-targets-briefing-150722.pdf

Table 45 Iron and Steel

Input	Value	Description
Annual Demand	11.2 Mt	Includes both domestic production (5.6Mt) and imports of finished and semi-finished products (5.6Mt). In 2023.
Baseline Emissions (kgCO2e/kgcH3OH)	1.83kgCO2e/kg Steel	Average of UK and global emissions intensities. https://api.repository.cam.ac.uk/server/api/core/bitstreams/a258e211-c841-4037-970e-009f1eb98373/content
Alternative Emissions 1	0.72kgCO2e/kg steel	EAF using recycled steel based on UK Grid intensity (162gCO2e/kWh)
Alternative Emissions 2	0.067kgCO2e/kg Steel	EAF using recycled steel based on renewable electricity (15gCO2e/kWh)
Hydrogen Demand (kgH2/kgH2)	61.6kg/t Steel	https://www.sciencedirect.com/science/article/pii/S0360319924026624
Other Inputs	0.45 kwh/kg Steel	Electric Arc furnace energy requirement https://www.sciencedirect.com/science/article/pii/S0360319924026624
Min Viable unit (kg H2)	12kt	10% of one plant, Average for one plant in Germany - DRI only, 4TWh/yr https://www.sciencedirect.com/science/article/pii/S0196890421012280#s0040

Table 46 Long duration grid balancing

Input	Value	Description
Annual Demand	10TWh	Based on current long term storage capacity – it is expected that more will be required to cope with fluctuations in renewable electricity. CCC estimates 40TWh needed by 2035 [88]
Baseline Emissions (kgCO2e/kgcH3OH)	0.410kgCO2e/kWh	Best in class emissions CCGT [89]
Alternative Emissions 1	0.2kgCO2e/kWh	Based on pumped hydro – exists at scale currently. 80% efficiency. https://www.sciencedirect.com/science/article/pii/S2352152X23020911#s0175
Alternative Emissions 2	0.01875kgCO2e/kwh	
Hydrogen Demand (kgH2/kgH2)	0.05	60% efficiency fuel cell (Maximum from range 40-60% [90])
Other Inputs	NA	NA
Min Viable unit (kg H2)	630t	Based on size of existing salt caverns in the UK and expected hydrogen density possible.[91]

Table 47 Non-Road Machinery

Input	Value	Description
Annual Demand	890000 hrs<br>3.5Mtoe. 40, 800TWh	UK statistics on non-road mobile machinery energy and emissions in tables env0201 and env0102 include military aviation and shipping, which are not the intended applications so base instead on red diesel use which was 15% of diesel use in the UK [68].
Baseline Emissions (kgCO2e/kgcH3OH)	0.24kgCO2e/kWh	GHG conversion factors for emissions factor for diesel [8]
Alternative Emissions 2	0.2kgCO2e/kWh	Electrification instead, thermal basis, 80% efficiency [90]
Alternative Emissions 1	0.01875kgCO2e/kwh	
Hydrogen Demand (kgH2/kgH2)	0.03 kgH2/kWh	Direct combustion, based on energy content.
Other Inputs	0	
Min Viable unit (kg H2)	29.2t	Current example of station capacity = 80kgH2/day [92]

Table 48 Bio-gas Upgrading

Input	Value	Description
Annual Demand	6.8 TWh	Amount of biomethane injected into the grid in 2022, assume as a baseline that no upgrading occurs.
Baseline Emissions ($kgCO_2e$/kWh biomethane)	0.32	Biogas 50-70% methane, 30-50% CO_2e, released to atmosphere if biogas is not upgraded [93]. Energy content of biomethane
Alternative Emissions	NA	NA
Hydrogen Demand (kgH_2/kgH_2)	0.07kgH_2/$kgCH_4$	Based on total methane output from biogas upgrading (Chemical Formula).
Other Inputs	Direct emissions combustion: 0.18$kgCO_2e$/kWh	Basis from production biogas to combustion natural gas.
Min Viable unit (kg H_2)	0	Upgrading occurs to some extent already. Replace existing.

Table 49 Long distance trucks and coaches

Input	Value	Description
Annual Demand	175 billion tkm	Transport Statistics UK domestic freight by road [64]
Baseline Emissions ($kgCO_2e$/$kgcH_3OH$)	0.119 $kgCO_2e$/tkm	GHG Conversion Factors 2022. Average all HGVs, 50% laden [8]
Alternative Emissions	0.009375 $kgCO_2e$/tkm (Renewable power)<br><br>0.10 $kgCO_2e$/tkm (Current Grid)	Based on GHG Conversion factors for diesel truck requires 0.5kWh/tkm. Battery 80% efficient (use of pantograph is an alternative, which would have higher efficiency).
Hydrogen Demand (kgH_2/kgH_2)	0.00687kgH_2/tkm	0.055kgH_2/vkm [76]
Other Inputs	0	
Min Viable unit (kg H_2)	29.2t	Current example of station capacity = 80kgH_2/day [92]

Table 50 High Temperature Heat

Input	Value	Description
Annual Demand	20 TWh	Based on 2019 Energy Consumption Data, use of Gas and Oil [94]. High temp process
Baseline Emissions (kgCO2e/kgcH3OH)	0.20264kgCO2e/kWh	GHG conversion factors, methane combustion, net CV [8]
Alternative Emissions	NA	NA
Hydrogen Demand (kgH2/kgH2)	0.03kgH2/kWh	Thermal basis, LHV hydrogen 33.3kWh/kgH2
Other Inputs	0	
Min Viable unit (kg H2)	2.7kt	Average demand for a glass container facility = 0.09TWh/yr [95]

Table 51 Commercial Heating

Input	Value	Description
Annual Demand	87 TWh	Based on 2019 Energy Consumption Data, use of Gas and Oil [71]. Space heating, water heating in service industry
Baseline Emissions (kgCO2e/kgcH3OH)	0.20264kgCO2e/kWh	GHG conversion factors, methane combustion, net CV
Alternative Emissions	0.005	Air Source Heat pump, COP 3 [96]
Hydrogen Demand (kgH2/kgH2)	0.03kgH2/kWh	Thermal basis, LHV 33.3kWh/kgH2
Other Inputs	0	
Min Viable unit (kg H2)	648kg	Average size of office ~4000sqft, average heating demand 60kWh/m2 = 21600kWh [97]

Table 52 Remote and Rural Trains

Input	Value	Description
Annual Demand	20 billion passenger km	https://assets.nationalrail.co.uk/e8xgegruud3g/1taBuYnOXlFeXr08eukGFn/6364913d37d3aff73a2d698c063932f9/Campaign_for_Better_Transport_-_RDG_Project_Methodology_v2.pdf
Baseline Emissions (kgCO2e/kgcH3OH)	0.106 kgCO2e /passenger km	https://assets.nationalrail.co.uk/e8xgegruud3g/1taBuYnOXlFeXr08eukGFn/6364913d37d3aff73a2d698c063932f9/Campaign_for_Better_Transport_-_RDG_Project_Methodology_v2.pdf
Alternative Emissions	0.0024kgCO2e/pkm	Hugh paper 0.16kWh/pkm (weighted average of all types)
Hydrogen Demand (kgH2/kgH2)	0.0078kgH2/pkm	Fuel cell 60% efficient
Other Inputs	NA	**NA**
Min Viable unit (kg H2)	327t	15,000 vehicles (includes carriages), assume 6 carriages per train, 19% diesel, each one does an average of 42 million pkm. Min unit = one train. /yrhttps://dataportal.orr.gov.uk/statistics/infrastructure-and-emissions/rail-infrastructure-and-assets/

Table 53 Vans

Input	Value	Description
Annual Demand	57.8 billion vehicle miles	https://www.gov.uk/government/statistics/road-traffic-estimates-in-great-britain-2023/road-traffic-estimates-in-great-britain-2023-traffic-in-great-britain-by-vehicle-type
Baseline Emissions (kgCO2e/kgcH3OH)	0.40273kgCO2e/vkm	GHG Conversion factor, van
Alternative Emissions	8.5e-3kgCO2e/vkm	0.57kWh/km https://www.sciencedirect.com/science/article/pii/S0306261920316810
Hydrogen Demand (kgH2/kgH2)	0.028kgH2/km	Fuel cell, 60% efficient
Other Inputs	NA	NA
Min Viable unit (kg H2)	29.2t	Current example of station capacity = 80kgH2/day [92]

Table 54 Metro Trains and Buses (Buses)

Input	Value	Description
Annual Demand	24 billion passenger km	Tsgb0101
Baseline Emissions (kgCO2e/kgcH3OH)	0.10846 kgCO2e/passengerkm	GHG conversion factor, average local bus 2024
Alternative Emissions	1.86kwh/vkm<br><br>0.0024kgCO2e/pkm	124–248 kWh/100 km , occupancy 11.3. 0.16kWh/pkm *https://www.sciencedirect.com/science/article/pii/S0360544217301081* https://www.gov.uk/government/statistical-data-sets/bus-statistics-data-tables
Hydrogen Demand (kgH2/kgH2)	7.8*10-3kgH2/pkm	Fuel cell 60% efficient
Other Inputs	NA	NA
Min Viable unit (kg H2)	29.2t	Current example of station capacity = 80kgH2/day [92]

Table 55 2 and 3 wheelers

Input	Value	Description
Annual Demand	2.9 billion vehicle miles	https://www.gov.uk/government/statistics/road-traffic-estimates-in-great-britain-2023/road-traffic-estimates-in-great-britain-2023-traffic-in-great-britain-by-vehicle-type
Baseline Emissions (kgCO2e/kgcH3OH)	0.11367kgCO2e/km	GHG conversion factor, average bike.
Alternative Emissions	4.2gCO2e/vkm	0.228kWh/km https://www.sciencedirect.com/science/article/pii/S2210670718318031#:~:text=The%20energy%20consumption%20of%20the,energy%20than%20the%20gasoline%20motorcycle
Hydrogen Demand (kgH2/kgH2)	0.0136kgH2/vkm	Combustion so use fuel demand based on GHG conversion factors – emissions factor assuming petrol. 0.45kWh required.
Other Inputs	NA	NA
Min Viable unit (kg H2)	29.2t	Current example of station capacity = 80kgH2/day [92]

Table 56 Cars and Taxis

Input	Value	Description
Annual Demand	251.3 billion vehicle miles	https://www.gov.uk/government/statistics/road-traffic-estimates-in-great-britain-2023/road-traffic-estimates-in-great-britain-2023-traffic-in-great-britain-by-vehicle-type
Baseline Emissions (kgCO2e/kgcH3OH)	0.16984kgCO2e/km	GHG conversion factor, average car
Alternative Emissions	2.85gCO2e/vkm	https://www.sciencedirect.com/science/article/pii/S0306261920316810
Hydrogen Demand (kgH2/kgH2)	0.01kgh2/vkm	https://www.sciencedirect.com/science/article/pii/S0306261920316810
Other Inputs	NA	NA
Min Viable unit (kg H2)	29.2t	Current example of station capacity = 80kgH2/day [92]

Table 57 Mid/Low Temp Heating

**Input**	**Value**	**Description**
Annual Demand	49 TWh	Based on 2019 Energy Consumption Data, use of Gas and Oil [94]. Low temp process + space heating
Baseline Emissions (kgCO2e/kgcH3OH)	0.20264kgCO2e/kWh	GHG conversion factors, methane combustion, net CV
Alternative Emissions	0.005kgCO2e/kWh	Heat pump with COP =3
Hydrogen Demand (kgH2/kgH2)	0.03kgH2/kg	Thermal basis
Other Inputs	NA	NA
Min Viable unit (kg H2)	1.2kt	Same energy capacity as a large industrial heat pump. (Number of houses and average use per house) https://www.mvv.de/journalisten/pressemitteilungen/detail/mvv-nimmt-ihre-erste-flusswaermepumpe-in-mannheim-in-betrieb

Table 58 Domestic Heating

**Input**	**Value**	**Description**
Annual Demand	283 TWh	Residential Energy use – space heating and water heating [94]
Baseline Emissions (kgCO2e/kgcH3OH)	0.20264kgCO2e/kWh	GHG conversion factors, methane combustion, net CV
Alternative Emissions	0.005kgCO2e/kWh	Heat pump with COP =3, renewable energy 15gCO2e/kWh
Hydrogen Demand (kgH2/kgH2)	0.03kgH2/kg	Thermal basis
Other Inputs	NA	NA
Min Viable unit (kg H2)	345kg	isolated house gas - average consumption of NG 11500kWh/yr/household<br><br>https://researchbriefings.files.parliament.uk/documents/CBP-9491/CBP-9491.pdf

3. Results

Additional results to those presented in the paper are discussed in this section, to provide further insights. The insights discussed include the optimal supply chains and impact of transmission for delivery of ammonia and methanol based on the supply chains studied, the results of the Sobol Analysis, the maximum emissions abatement potential compared to electrification based on current grid emissions, the range in emissions abatement potential if non-optimal supply chains are used and the emissions abatement potential per unit of renewable energy for hydrogen applications compared to direct electrification.

3.1. Emissions Intensity of Methanol Supply Chains

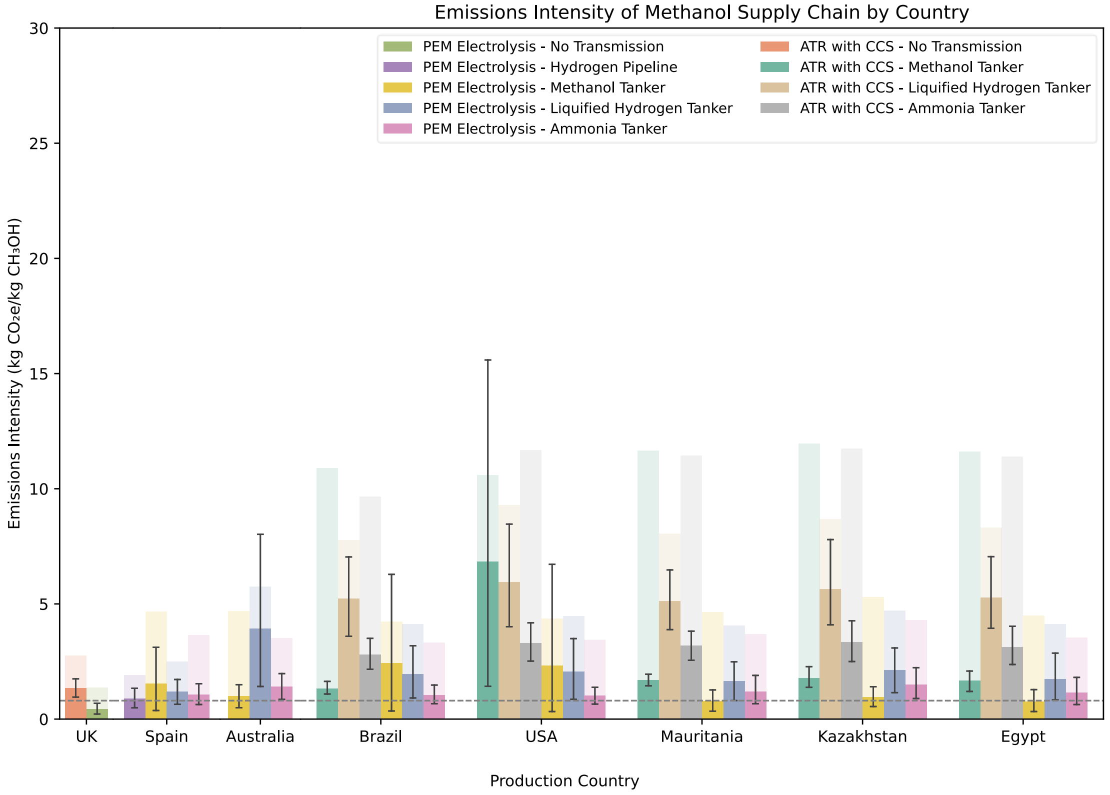

Figure 5 Emissions intensity of both domestic and imported methanol supply chains for the lowest emissions supply chain based on the production country, main transmission vector, and production method. The uncertainty bars show the maximum and minimum bounds of the Monte Carlo analysis for each pathway. The translucent bars show the emissions intensity of the supply chain with the highest emissions that has the same origin, production method, and main transmission vector. The emissions intensity bars are compared to the emissions intensity of current methanol production (0.9 kg CO2e/ kg H2).

Figure 5 shows that, as expected, transmission of methanol via tanker is the optimal pathway for most global supply chains if methanol is desired. The emissions could be lower than current methanol production for both electrolytic green methanol production in the UK and the lowest emissions pathways from all countries. However, in some cases the emissions are drastically higher, such as if ATR with CCS is used as the production method for hydrogen.

3.2. Emissions Intensity of Ammonia Supply Chains

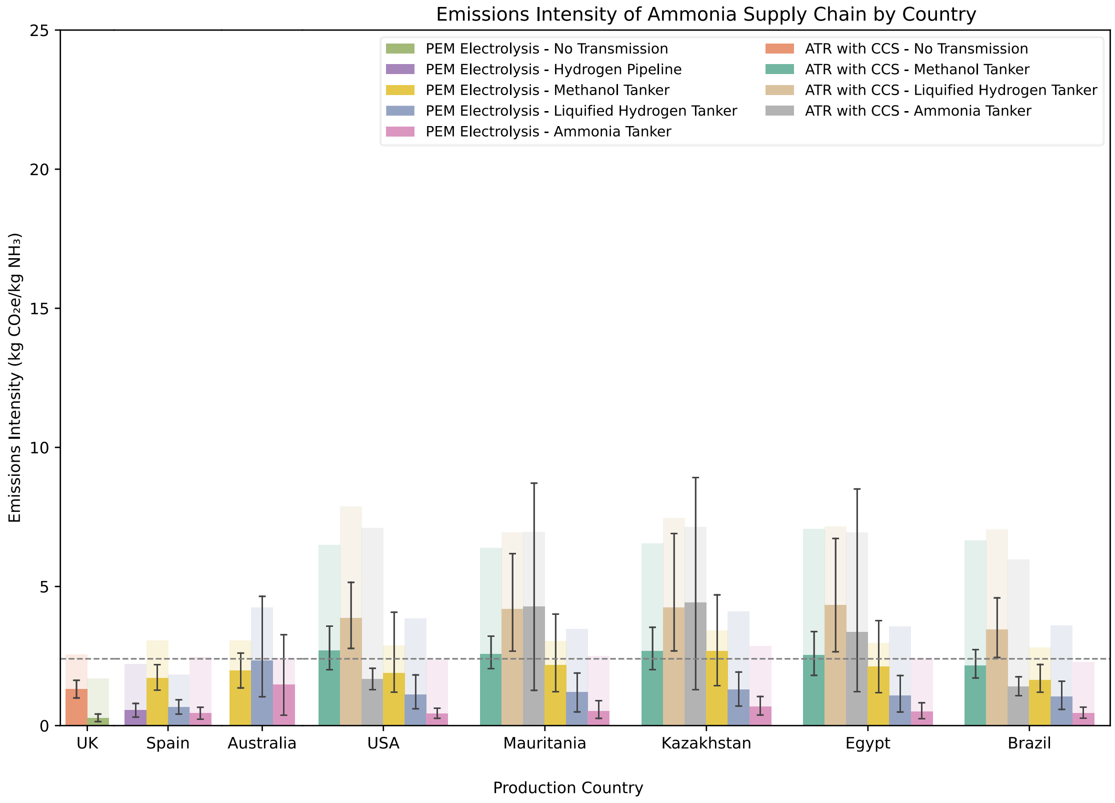

Figure 6 Emissions intensity of both domestic and imported ammonia supply chains for the lowest emissions supply chain based on the production country, main transmission vector, and production method. The uncertainty bars show the maximum and minimum bounds of the Monte Carlo analysis for each pathway. The translucent bars show the emissions intensity of the supply chain with the highest emissions that has the same origin, production method, and main transmission vector. The emissions intensity bars are compared to the emissions intensity of current ammonia production (2.9 kg CO2e/ kg H2).

Figure 6 shows that, as expected, transmission of ammonia via tanker is the optimal pathway for all global supply chains if ammonia is desired. The emissions are lower than current ammonia production for both electrolytic green ammonia produced in the UK and international production. Transmission via liquified hydrogen could also allow reduced emissions in some cases.

3.3. Impact of Decarbonised Transport

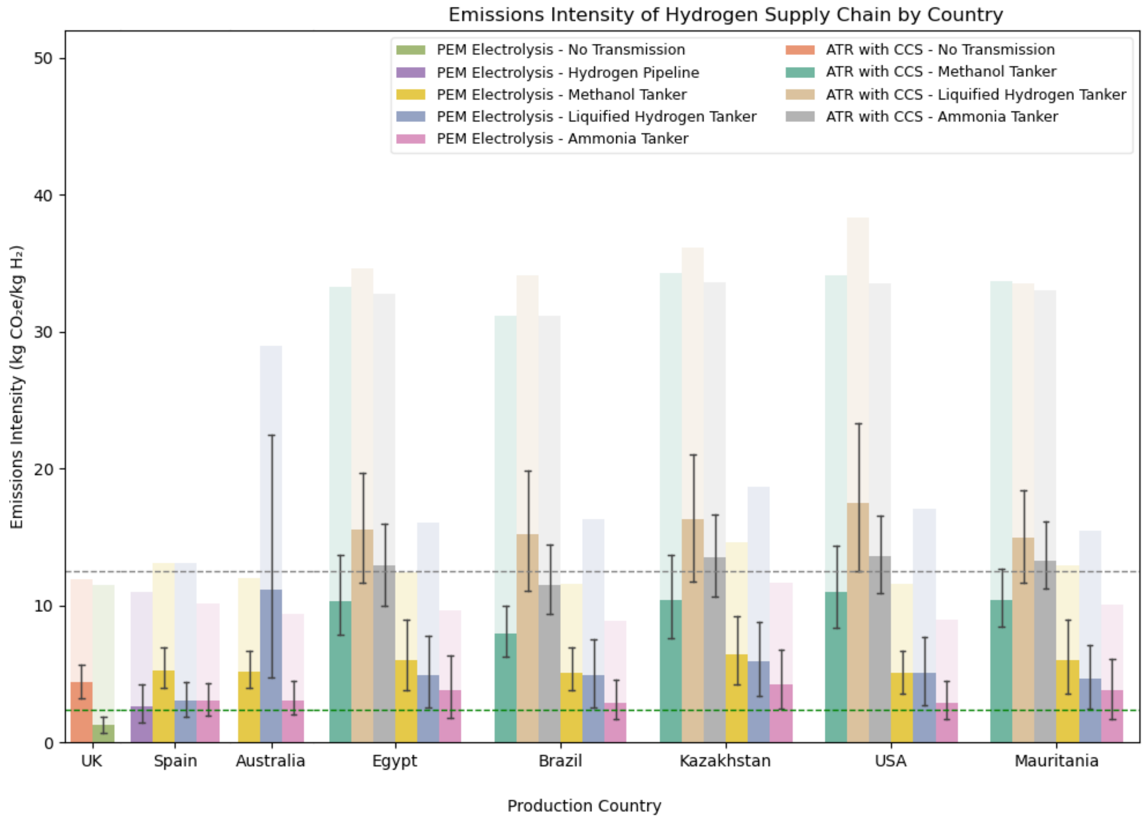

Figure 7 Emissions intensity of both domestic and imported hydrogen supply chains for the lowest emissions supply chain based on the production country, main transmission vector, and production method. The lighter shaded area denotes the average contribution from energy use in transmission, and the error bars show the full range of possible values if decarbonised transport options are available. The emissions intensity bars are compared to the emissions intensity of current hydrogen production (2.4 kg CO2e/ kg H2). In this figure no emissions are assumed from the use of fuels in transport.

Figure 7 shows that decarbonised shipping has most impact on supply chains which use liquified hydrogen as the shipping vector, but it would decrease the emissions intensity of all shipping pathways to some degree. This could enable hydrogen from countries including Brazil, USA and Mauritania to align with the UK Low Carbon Hydrogen Standard (2.4 kgCO2e/kg H2) but imports from Australia, Egypt and Kazakhstan are likely to exceed the standard even in the most optimistic scenarios. The largest reduction is seen in transmission via liquified hydrogen from Australia where there is a 25% reduction. The average decrease in emissions intensity of hydrogen seen across all supply chains that rely on shipping is 9%.

3.4. Sobol Analysis

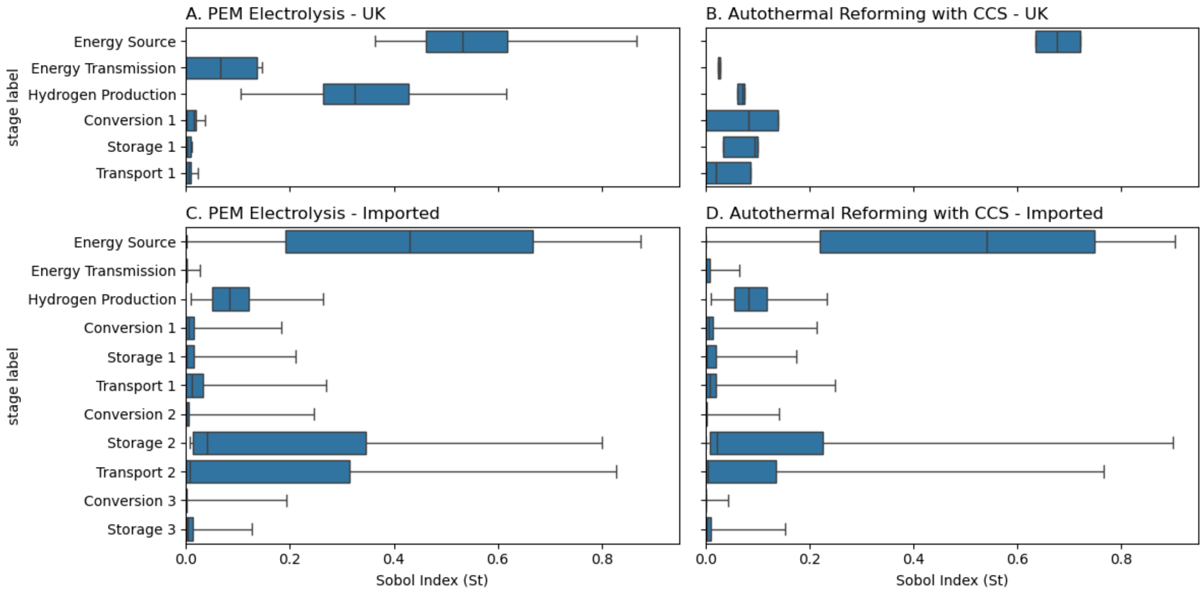

Figure 8 Sobol Indices showing the importance of each stage on the variance of the results for each supply chain configuration. The results are split by production method and whether production occurs within the UK or internationally.

In figure 8, the Sobol Indices suggest that for most supply chains the energy source variables cause the most variation in the emissions intensity of a given supply chain. These variables are the emissions intensity of the energy source, and the utilisation factor of electrolysis as it is linked to the energy source. The next most important stages are the hydrogen production impacts for production in the UK or storage and transmission for international production.

3.5. Emissions abatement potential versus current grid emissions

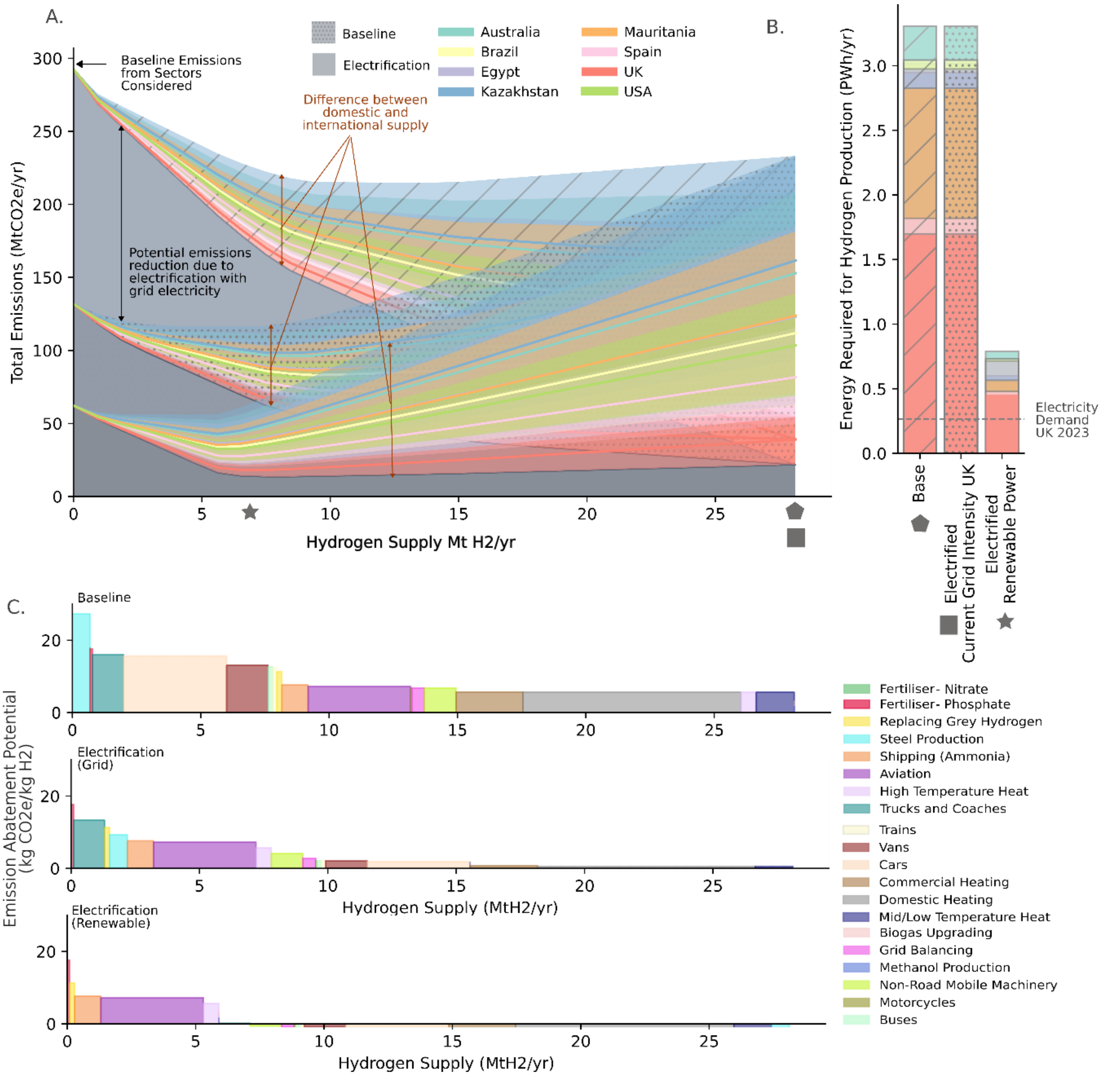

Figure 9 A. Total emissions in the UK from the sectors listed as a function of the amount of hydrogen available per year. It is assumed that the hydrogen can be supplied from the minimum emission hydrogen supply chain for each production country. The baseline scenario compares the hydrogen supply chain emission intensity to current emissions whereas in the electrification scenario all viable applications are first electrified, assuming an emissions intensity for renewable power of 15 gCO2e/ kWh. The regions display the uncertainty from the Monte Carlo analysis. The amount of hydrogen required for the minimum emission scenario is shown by the star symbol for electrification and pentagon for the baseline scenario. After the minimum emissions point the emissions begin to increase as hydrogen use has higher impacts than the alternative. B. The energy that would be required for hydrogen production for the baseline and electrification minimum emissions scenarios. C. The calculated emissions abatement potential and demand for each sector for the baseline and electrification scenarios based on the minimum emission pathway.

In figure 9, it is shown that electrifying all processes based on the current grid intensity would decrease emissions by over 100 Mt CO2e/a, but if low emission hydrogen is available emissions could further be reduced. However, this would require large amounts of renewable energy that could instead be used to decarbonise the grid further. The figure shows that for most applications the switch between electrification and hydrogen as the optimal solution occurs when the emissions intensity of electricity is between 15-160 g CO2e/kWh.

3.6. Maximum range of emissions abatement potential

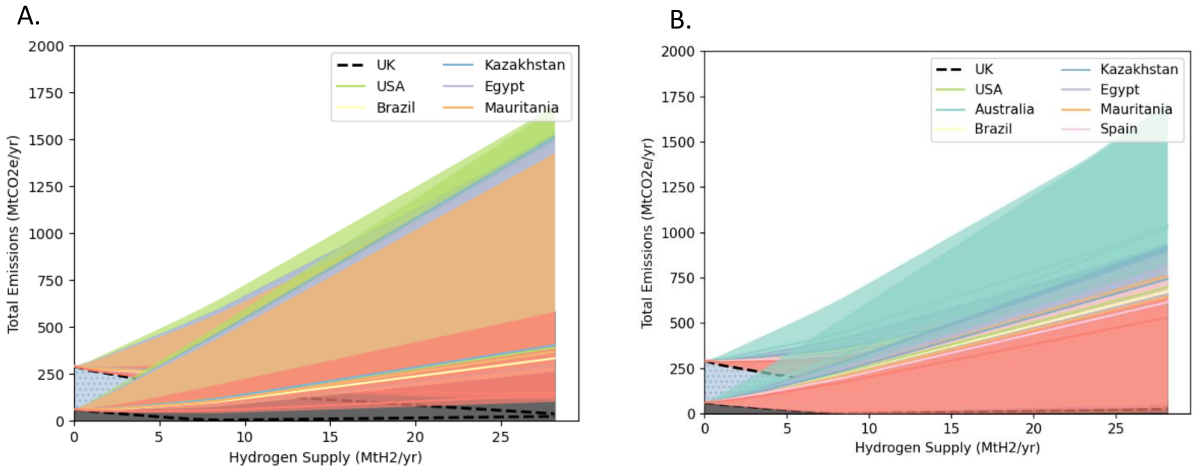

Figure 10 A. The range of total emissions depending on hydrogen supply based on use of ATR with CCS to produce hydrogen from all supply chains. B. The range of total emissions depending on hydrogen supply based on use of PEM electrolysis to produce hydrogen from all supply chains.

Figures 10 A and B show that if the lowest emission supply chains are not used then emissions from the sectors considered could drastically increase through use of hydrogen. This is particularly true when considering ATR with CCS as the production method, or for transmission routes that are very long such as Australia to the UK. Standards need to be in place to ensure that this is avoided if the use of hydrogen is increased to avoid increasing global emissions. Even if hydrogen is produced in the UK via PEM electrolysis there are some supply chains that would allow minimal emissions reductions.

3.7. Emissions abatement potential per unit of renewable energy

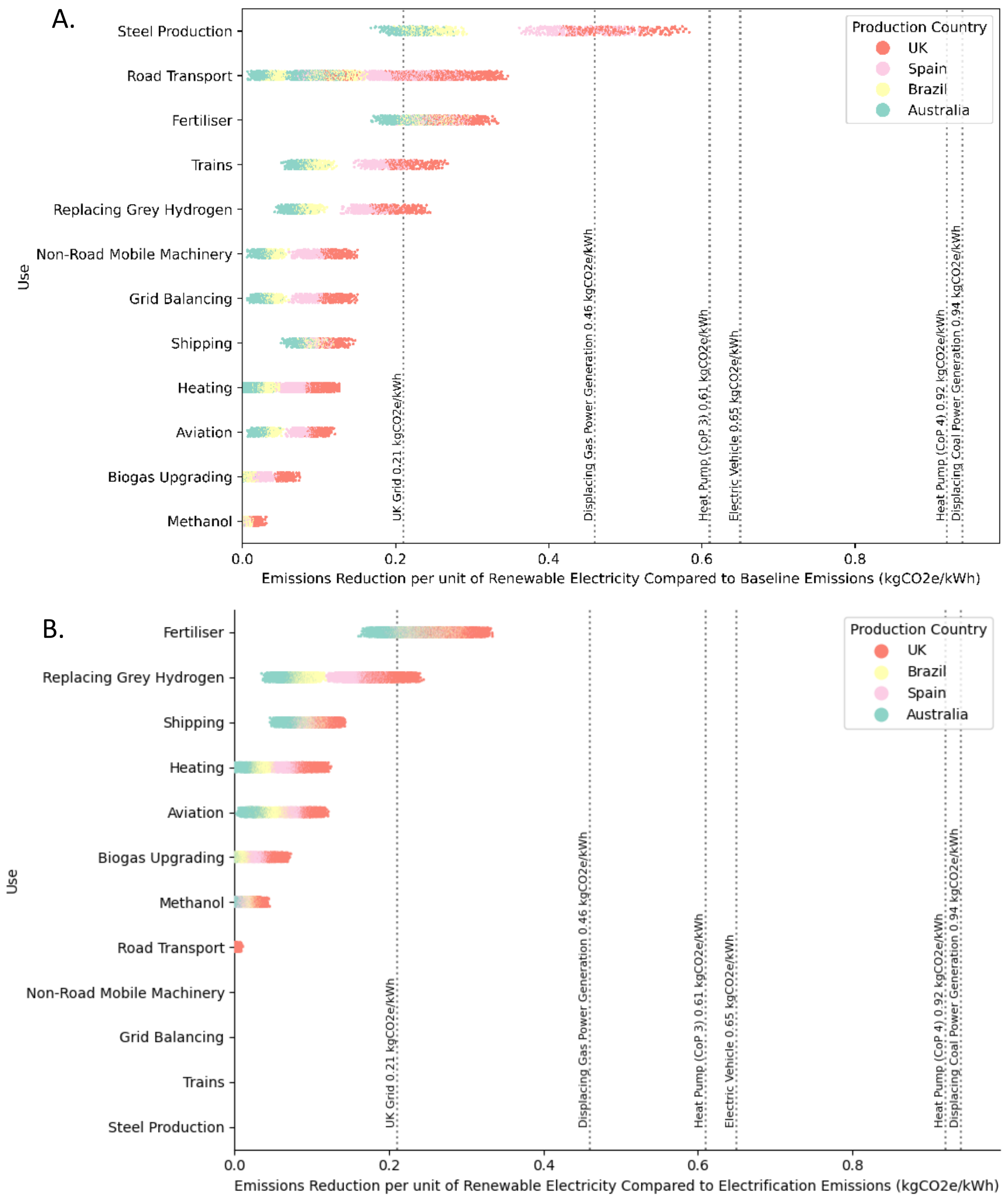

Figure 11 A. The emissions reduction potential of renewable energy used to produce hydrogen compared to baseline emissions. Vertical lines show the emission reduction potential of direct electrification applications to show the trade-off between using electricity directly and using electricity to produce hydrogen. The points represent the results for each supply chain configuration including the Monte Carlo analysis results. B. The emissions reduction potential of renewable energy used to produce hydrogen compared to electrification with renewable electricity where possible.

Though for some sectors there are few other decarbonisation options other than low emission hydrogen, such as where it is a feedstock for the desired product, it may not be the case that hydrogen production should be prioritised. In figures 11 A and B, it is shown that using renewable energy to displace coal power generation, electric vehicles, and heating using heat pumps would reduce emissions more per unit of energy than any use of hydrogen. Therefore, where possible these uses should be prioritised above hydrogen implementation. Use of hydrogen for primary steel otherwise would mitigate the most emissions per unit of renewable energy versus current emissions, and production of fertilisers is the priority if all sectors have been electrified.

References

[1] A. Bennett and A. C. Serrenho, "A systematic comparison of the energy and emissions intensity of hydrogen production pathways in the United Kingdom," *Int J Hydrogen Energy*, vol. 89, pp. 364–374, Nov. 2024, doi: 10.1016/J.IJHYDENE.2024.09.170.

[2] M. Sterner *et al.*, "19 Import options for green hydrogen and derivatives - An overview of efficiencies and technology readiness levels," *Int J Hydrogen Energy*, vol. 90, pp. 1112–1127, Nov. 2024, doi: 10.1016/J.IJHYDENE.2024.10.045.

[3] "Hydrogen Production and Infrastructure Projects Database - Data product - IEA." Accessed: Dec. 16, 2024. [Online]. Available: https://www.iea.org/data-and-statistics/data-product/hydrogen-production-and-infrastructure-projects-database

[4] V. Kouloumpis and A. Azapagic, "A model for estimating life cycle environmental impacts of offshore wind electricity considering specific characteristics of wind farms," *Sustain Prod Consum*, vol. 29, pp. 495–506, Jan. 2022, doi: 10.1016/J.SPC.2021.10.024.

[5] S. Irving, A. Bonou, A. Laurent, and S. I. Olsen, "General rights Life cycle assessment of onshore and offshore wind energy-from theory to application Life cycle assessment of onshore and offshore wind energy: from theory to application", doi: 10.1016/j.apenergy.2016.07.058.

[6] J. H. C. Bosmans, L. C. Dammeier, and M. A. J. Huijbregts, "Greenhouse gas footprints of utility-scale photovoltaic facilities at the global scale," *Environmental Research Letters*, vol. 16, no. 9, p. 094056, Sep. 2021, doi: 10.1088/1748-9326/AC1DF9.

[7] V. Scott, "The Sixth Carbon Budget: The UK's path to Net Zero," 2020. [Online]. Available: https://www.theccc.org.uk/publication/sixth-carbon-budget/

[8] Department for Business Energy & Industrial Strategy, "GHG Conversion Factors 2022 Condensed Set." Accessed: Dec. 08, 2022. [Online]. Available: https://www.gov.uk/government/publications/greenhouse-gas-reporting-conversion-factors-2022

[9] North Sea Transition Authority, "Natural Gas Carbon Footprint Analysis." Accessed: Jan. 30, 2024. [Online]. Available: https://www.nstauthority.co.uk/the-move-to-net-zero/net-zero-benchmarking-and-analysis/natural-gas-carbon-footprint-analysis/

[10] L. C. Dammeier, J. H. C. Bosmans, and M. A. J. Huijbregts, "Variability in greenhouse gas footprints of the global wind farm fleet," *J Ind Ecol*, vol. 27, no. 1, pp. 272–282, Feb. 2023, doi: 10.1111/JIEC.13325.

[11] "Australia: power sector carbon emission forecast 2050 | Statista." Accessed: Dec. 19, 2024. [Online]. Available: https://www.statista.com/statistics/1184328/power-sector-emissions-outlook-australia/

[12] "PER CAPITA GREENHOUSE GAS (GHG) EMISSIONS ABOVE G20 AVERAGE RECENT DEVELOPMENTS".

[13] "Contrasting Upstream Emissions in Northwest Australia Offshore | S&P Global." Accessed: Oct. 01, 2024. [Online]. Available: https://www.spglobal.com/commodityinsights/en/ci/research-analysis/contrasting-upstream-emissions-in-northwest-australia-offshore.html

[14] "Electricity Data Explorer | Open Source Global Electricity Data | Ember." Accessed: Oct. 01, 2024. [Online]. Available: https://ember-climate.org/data/data-tools/data-explorer/

[15] J. Littlefield, S. Rai, and T. J. Skone, "Life Cycle GHG Perspective on U.S. Natural Gas Delivery Pathways," *Environ Sci Technol*, vol. 56, no. 22, pp. 16033–16042, Nov. 2022, doi: 10.1021/ACS.EST.2C01205/ASSET/IMAGES/LARGE/ES2C01205_0007.JPEG.

[16] "Spain power sector carbon intensity outlook 2050 | Statista." Accessed: Dec. 19, 2024. [Online]. Available: https://www.statista.com/statistics/1190075/carbon-intensity-outlook-of-spain/

[17] "Mauritania | Climate Promise." Accessed: Dec. 19, 2024. [Online]. Available: https://climatepromise.undp.org/what-we-do/where-we-work/mauritania

[18] I. - International Energy Agency, "Emissions from Oil and Gas Operations in Net Zero Transitions A World Energy Outlook Special Report on the Oil and Gas Industry and COP28", Accessed: Oct. 01, 2024. [Online]. Available: www.iea.org

[19] "Global Natural Gas Production | World gas natural statistics | Enerdata." Accessed: Oct. 01, 2024. [Online]. Available: https://yearbook.enerdata.net/natural-gas/world-natural-gas-production-statistics.html

[20] "2050 Projections for CO2 Intensity of Electricity Generation | Enerdata." Accessed: Dec. 19, 2024. [Online]. Available: https://eneroutlook.enerdata.net/forecast-world-co2-intensity-of-electricity-generation.html

[21] "GHG intensity of offshore Brazil production in 2022 | S&P Global." Accessed: Oct. 01, 2024. [Online]. Available: https://www.spglobal.com/commodityinsights/en/ci/research-analysis/ghg-intensity-of-offshore-brazil-production-in-2022.html

[22] "Executive summary – Kazakhstan 2022 – Analysis - IEA." Accessed: Dec. 19, 2024. [Online]. Available: https://www.iea.org/reports/kazakhstan-2022/executive-summary

[23] "Egypt | Climate Promise." Accessed: Dec. 19, 2024. [Online]. Available: https://climatepromise.undp.org/what-we-do/where-we-work/egypt

[24] "CO2 emissions intensity of electricity generation in the Announced Pledges Scenario, 2022-2030 – Charts – Data & Statistics - IEA." Accessed: Oct. 01, 2024. [Online]. Available: https://www.iea.org/data-and-statistics/charts/co2-emissions-intensity-of-electricity-generation-in-the-announced-pledges-scenario-2022-2030

[25] "Greenhouse gas reporting: conversion factors 2024 - GOV.UK." Accessed: Oct. 01, 2024. [Online]. Available: https://www.gov.uk/government/publications/greenhouse-gas-reporting-conversion-factors-2024

[26] F. Mendler, J. Fragoso Garcia, C. Kleinschmitt, and C. Voglstätter, "Global optimization of capacity ratios between electrolyser and renewable electricity source to minimize levelized cost of green hydrogen," *Int J Hydrogen Energy*, vol. 82, pp. 986–993, Sep. 2024, doi: 10.1016/J.IJHYDENE.2024.07.320.

[27] K. Bareiß, C. de la Rua, M. Möckl, and T. Hamacher, "Life cycle assessment of hydrogen from proton exchange membrane water electrolysis in future energy systems," *Appl Energy*, vol. 237, pp. 862–872, Mar. 2019, doi: 10.1016/J.APENERGY.2019.01.001.

[28] Ansys, “Ansys Granta EduPack,” 2023.

[29] “Home | ecoQuery.” Accessed: Dec. 17, 2024. [Online]. Available: https://ecoquery.ecoinvent.org/3.9.1/cutoff/search

[30] S. Krishnan, B. Corona, G. J. Kramer, M. Junginger, and V. Koning, “Prospective LCA of alkaline and PEM electrolyser systems,” *Int J Hydrogen Energy*, vol. 55, pp. 26–41, Feb. 2024, doi: 10.1016/J.IJHYDENE.2023.10.192.

[31] EPRI, “Water Electrolyzer Stack Degradation.” Accessed: Oct. 02, 2024. [Online]. Available: https://www.epri.com/research/sectors/lcri/research-results/3002025148

[32] Potsdam Institute for Climate Research, “Price of Hydrogen: CAPEX Data.” Accessed: Aug. 01, 2023. [Online]. Available: https://h2.pik-potsdam.de/H2Dash/#section-visualisations

[33] J. Cooper, L. Dubey, S. Bakkaloglu, and A. Hawkes, “Hydrogen emissions from the hydrogen value chain-emissions profile and impact to global warming,” *Science of The Total Environment*, vol. 830, p. 154624, Jul. 2022, doi: 10.1016/J.SCITOTENV.2022.154624.

[34] N. Gerloff, “Comparative Life-Cycle-Assessment analysis of three major water electrolysis technologies while applying various energy scenarios for a greener hydrogen production,” *J Energy Storage*, vol. 43, p. 102759, Nov. 2021, doi: 10.1016/J.EST.2021.102759.

[35] S. Krishnan *et al.*, “Present and future cost of alkaline and PEM electrolyser stacks,” *Int J Hydrogen Energy*, vol. 48, no. 83, pp. 32313–32330, Oct. 2023, doi: 10.1016/j.ijhydene.2023.05.031.

[36] A. O. Oni, K. Anaya, T. Giwa, G. Di Lullo, and A. Kumar, “Comparative assessment of blue hydrogen from steam methane reforming, autothermal reforming, and natural gas decomposition technologies for natural gas-producing regions,” *Energy Convers Manag*, vol. 254, p. 115245, Feb. 2022, doi: 10.1016/J.ENCONMAN.2022.115245.

[37] P. L. Spath and M. K. Mann, “Life Cycle Assessment of Hydrogen Production via Natural Gas Steam Reforming”, Accessed: Jan. 31, 2024. [Online]. Available: http://www.doe.gov/bridge

[38] EcoInvent v3.1, “chemical factory construction, organics - RER - chemical factory, organics.” Accessed: Feb. 02, 2024. [Online]. Available: https://ecoquery.ecoinvent.org/3.10/cutoff/dataset/8400/documentation

[39] NETL, “NETL Life Cycle Inventory Data – Unit Process: Autothermal Reforming with CCS.” Accessed: Oct. 02, 2024. [Online]. Available: http://www.netl.doe.gov/LCA

[40] G. Zang, E. J. Graham, and D. Mallapragada, “H2 production through natural gas reforming and carbon capture: A techno-economic and life cycle analysis comparison,” *Int J Hydrogen Energy*, vol. 49, pp. 1288–1303, Jan. 2024, doi: 10.1016/J.IJHYDENE.2023.09.230.

[41] IEA, “Comparison of the emissions intensity of different hydrogen production routes.” Accessed: Feb. 19, 2024. [Online]. Available: https://www.iea.org/data-and-statistics/charts/comparison-of-the-emissions-intensity-of-different-hydrogen-production-routes-2021

[42] T. Terlouw, K. Treyer, C. Bauer, and M. Mazzotti, “Life Cycle Assessment of Direct Air Carbon Capture and Storage with Low-Carbon Energy Sources,” *Environ Sci Technol*, vol. 55, no. 16,

pp. 11397–11411, Aug. 2021, doi: 10.1021/ACS.EST.1C03263/SUPPL_FILE/ES1C03263_SI_002.ZIP.

[43] S. Z. Al Ghafri *et al.*, “Hydrogen liquefaction: a review of the fundamental physics, engineering practice and future opportunities,” *Energy Environ Sci*, vol. 15, no. 7, pp. 2690–2731, Jul. 2022, doi: 10.1039/D2EE00099G.

[44] K. de Kleijne *et al.*, “Worldwide greenhouse gas emissions of green hydrogen production and transport,” *Nature Energy 2024*, pp. 1–14, Jun. 2024, doi: 10.1038/s41560-024-01563-1.

[45] C. Wulf *et al.*, “Life Cycle Assessment of hydrogen transport and distribution options,” *J Clean Prod*, vol. 199, pp. 431–443, Oct. 2018, doi: 10.1016/J.JCLEPRO.2018.07.180.

[46] J. Osorio-Tejada, N. N. Tran, and V. Hessel, “Techno-environmental assessment of small-scale Haber-Bosch and plasma-assisted ammonia supply chains,” *Science of The Total Environment*, vol. 826, p. 154162, Jun. 2022, doi: 10.1016/J.SCITOTENV.2022.154162.

[47] K. de Kleijne, H. de Coninck, R. van Zelm, M. A. J. Huijbregts, and S. V. Hanssen, “The many greenhouse gas footprints of green hydrogen,” *Sustain Energy Fuels*, vol. 6, no. 19, pp. 4383–4387, Sep. 2022, doi: 10.1039/D2SE00444E.

[48] EcoInvent v3.9.1, “ammonia production, steam reforming, liquid - RER w/o RU - ammonia, anhydrous, liquid.” Accessed: Feb. 06, 2024. [Online]. Available: https://ecoquery.ecoinvent.org/3.9.1/cutoff/dataset/23374/exchanges

[49] S. Sollai, A. Porcu, V. Tola, F. Ferrara, and A. Pettinau, “Renewable methanol production from green hydrogen and captured CO2: A techno-economic assessment,” *Journal of CO2 Utilization*, vol. 68, p. 102345, Feb. 2023, doi: 10.1016/J.JCOU.2022.102345.

[50] F. M. Kanchiralla, S. Brynolf, E. Malmgren, J. Hansson, and M. Grahn, “Life-Cycle Assessment and Costing of Fuels and Propulsion Systems in Future Fossil-Free Shipping,” *Environ Sci Technol*, vol. 56, no. 17, pp. 12517–12531, Sep. 2022, doi: 10.1021/ACS.EST.2C03016,.

[51] “methanol factory construction - Global - methanol factory | ecoQuery.” Accessed: Oct. 04, 2024. [Online]. Available: https://ecoquery.ecoinvent.org/3.9.1/cutoff/dataset/567/documentation

[52] S. Zhang, Y. Liu, M. Zhang, Y. Ma, J. Hu, and Y. Qu, “Sustainable production of hydrogen with high purity from methanol and water at low temperatures,” *Nature Communications 2022 13:1*, vol. 13, no. 1, pp. 1–10, Sep. 2022, doi: 10.1038/s41467-022-33186-z.

[53] “pipeline construction, natural gas, long distance, high capacity, onshore - Global - pipeline, natural gas, long distance, high capacity, onshore | ecoQuery.” Accessed: Oct. 03, 2024. [Online]. Available: https://ecoquery.ecoinvent.org/3.9.1/cutoff/dataset/3496/documentation

[54] “About half of U.S. oil pipeline space is empty after boom time building spree | Reuters.” Accessed: Oct. 03, 2024. [Online]. Available: https://www.reuters.com/markets/commodities/about-half-us-oil-pipeline-space-is-empty-after-boom-time-building-spree-2021-12-16/

[55] C. Wong, M. R. Dann, and R. Wong, “Life Expectancy of Decommissioned Pipelines Under External Corrosion: Probabilistic Modeling,” *Proceedings of the Biennial International Pipeline Conference, IPC*, vol. 1, Jan. 2021, doi: 10.1115/IPC2020-9329.

[56] “Top 10 Longest Pipelines in the World: Oil and Gas Transportation Giants.” Accessed: Oct. 02, 2024. [Online]. Available: https://www.offshore-technology.com/features/worlds-longest-pipelines/

[57] “transport, pipeline, long distance, natural gas - Europe without Germany, the Netherlands, and Russia - transport, pipeline, long distance, natural gas | ecoQuery.” Accessed: Oct. 02, 2024. [Online]. Available: https://ecoquery.ecoinvent.org/3.9.1/cutoff/dataset/19116/documentation

[58] EcoInvent v3.9.1, “pipeline construction, natural gas, long distance, high capacity, offshore - GLO - pipeline, natural gas, long distance, high capacity, offshore.” Accessed: Feb. 05, 2024. [Online]. Available: https://ecoquery.ecoinvent.org/3.9.1/cutoff/dataset/1335/impact_assessment

[59] “The Pipeline - Nord Stream AG.” Accessed: Oct. 02, 2024. [Online]. Available: https://www.nord-stream.com/the-project/pipeline/

[60] P. Martin *et al.*, “A review of challenges with using the natural gas system for hydrogen,” *Energy Sci Eng*, 2024, doi: 10.1002/ESE3.1861.

[61] C. Tsiklios, M. Hermesmann, and T. E. Müller, “Hydrogen transport in large-scale transmission pipeline networks: Thermodynamic and environmental assessment of repurposed and new pipeline configurations,” *Appl Energy*, vol. 327, p. 120097, Dec. 2022, doi: 10.1016/J.APENERGY.2022.120097.

[62] “Baltic cable | Hitachi Energy.” Accessed: Oct. 31, 2024. [Online]. Available: https://www.hitachienergy.com/uk-ie/en/news-and-events/customer-success-stories/baltic-cable

[63] “transmission network construction, electricity, high voltage direct current subsea cable - Europe - transmission network, electricity, high voltage direct current subsea cable | ecoQuery.” Accessed: Oct. 03, 2024. [Online]. Available: https://ecoquery.ecoinvent.org/3.9.1/cutoff/dataset/26891/documentation

[64] “World’s longest subsea power cable connects Britain to Denmark | Q4 2023 Quarterly Report | Electric Insights.” Accessed: Oct. 03, 2024. [Online]. Available: https://reports.electricinsights.co.uk/q4-2023/worlds-longest-subsea-power-cable-connects-britain-to-denmark/

[65] “UK Power Networks’ Distribution Losses Strategy,” 2019.

[66] “50hertz.com > Grid > Grid developement > Concluded projects > Kontek 400-KV Grid Connection.” Accessed: Oct. 31, 2024. [Online]. Available: https://www.50hertz.com/en/Grid/Griddevelopement/Concludedprojects/Kontek400-KVGridConnection

[67] “transmission network construction, electricity, high voltage direct current land cable - Europe - transmission network, electricity, high voltage direct current land cable | ecoQuery.” Accessed: Oct. 03, 2024. [Online]. Available: https://ecoquery.ecoinvent.org/3.9.1/cutoff/dataset/26064/documentation

[68] “Top 16 Biggest LNG Ships of 2024.” Accessed: Oct. 30, 2024. [Online]. Available: https://www.marineinsight.com/types-of-ships/biggest-lng-ships/

[69] EcoInvent v3.9.1, “tanker production, for liquefied natural gas - GLO - tanker, for liquefied natural gas.” Accessed: Feb. 05, 2024. [Online]. Available: https://ecoquery.ecoinvent.org/3.9.1/cutoff/dataset/21885/documentation

[70] “MOL recycles its oldest LNG carrier - Offshore Energy.” Accessed: Oct. 30, 2024. [Online]. Available: https://www.offshore-energy.biz/mol-recycles-its-oldest-lng-carrier/

[71] “Hydrogen trade: Can shipping deliver a global hydrogen market? - Hydrogen Science Coalition.” Accessed: Dec. 19, 2024. [Online]. Available: https://h2sciencecoalition.com/blog/hydrogen-trade-can-shipping-deliver-a-global-hydrogen-market/

[72] “IEA G20 Hydrogen report: Assumptions”.

[73] Q. Song *et al.*, “A comparative study on energy efficiency of the maritime supply chains for liquefied hydrogen, ammonia, methanol and natural gas,” *Carbon Capture Science & Technology*, vol. 4, p. 100056, Sep. 2022, doi: 10.1016/J.CCST.2022.100056.

[74] J. Hampp, M. Düren, and T. Brown, “Import options for chemical energy carriers from renewable sources to Germany,” *PLoS One*, vol. 18, no. 2, p. e0262340, Feb. 2023, doi: 10.1371/JOURNAL.PONE.0281380.

[75] “Long haul semi-trailer truck - Liquefied natural gas (LNG) | NATIONAL ROAD COMMITTEE.” Accessed: Dec. 19, 2024. [Online]. Available: https://www.cnr.fr/en/espace-standard/18

[76] D. Ainalis, C. Thorne, and D. Cebon, “Technoeconomic comparison of an electric road system and hydrogen for decarbonising the UK’s long-haul road freight,” *Research in Transportation Business & Management*, vol. 48, p. 100914, Jun. 2023, doi: 10.1016/J.RTBM.2022.100914.

[77] C. Moran, P. Deane, S. Yousefian, and R. F. D. Monaghan, “The hydrogen storage challenge: Does storage method and size affect the cost and operational flexibility of hydrogen supply chains?,” *Int J Hydrogen Energy*, vol. 52, pp. 1090–1100, Jan. 2024, doi: 10.1016/J.IJHYDENE.2023.06.269.

[78] IEA, “IEA G20 Hydrogen report: Assumptions.” Accessed: Oct. 29, 2024. [Online]. Available: https://iea.blob.core.windows.net/assets/a02a0c80-77b2-462e-a9d5-1099e0e572ce/IEA-The-Future-of-Hydrogen-Assumptions-Annex.pdf

[79] G. Palmer, A. Roberts, A. Hoadley, R. Dargaville, and D. Honnery, “Life-cycle greenhouse gas emissions and net energy assessment of large-scale hydrogen production via electrolysis and solar PV,” *Energy Environ Sci*, vol. 14, no. 10, pp. 5113–5131, Oct. 2021, doi: 10.1039/D1EE01288F.

[80] L. D. D. Harvey, “A bottom-up assessment of recent (2016–20) energy use by the global iron and steel industry constrained to match a top-down (International Energy Agency) assessment,” *Energy*, vol. 293, p. 130675, Apr. 2024, doi: 10.1016/J.ENERGY.2024.130675.

[81] A. Agostini *et al.*, “Role of hydrogen tanks in the life cycle assessment of fuel cell-based auxiliary power units,” *Appl Energy*, vol. 215, pp. 1–12, Apr. 2018, doi: 10.1016/J.APENERGY.2018.01.095.

[82] S. Kolb, J. Müller, N. Luna-Jaspe, and J. Karl, “Renewable hydrogen imports for the German energy transition – A comparative life cycle assessment,” *J Clean Prod*, vol. 373, p. 133289, Nov. 2022, doi: 10.1016/J.JCLEPRO.2022.133289.

[83] Liebreich Associates, “The Clean Hydrogen Ladder .” Accessed: Aug. 24, 2023. [Online]. Available: https://www.liebreich.com/the-clean-hydrogen-ladder-now-updated-to-v4-1/

[84] H. Vatankhah Ghadim, R. A. M. Peer, E. G. Read, and J. Haas, “How much hydrogen could we need in New Zealand? Understanding the diverse hydrogen applications and their regional mapping,” *J R Soc N Z*, pp. 1–20, Jun. 2024, doi: 10.1080/03036758.2024.2365306.

[85] “Nitrogen fertilisers | AHDB.” Accessed: Oct. 22, 2024. [Online]. Available: https://ahdb.org.uk/nitrogen-fertiliser-market-outlook

[86] “The Economic and Environmental Impact of the Historic and Classic Motor Industry in the UK A CEBR Report for HERO-ERA Authorship and acknowledgements,” 2020.

[87] National Grid, “National Grid Electricity Transmission.” Accessed: Dec. 17, 2024. [Online]. Available: https://www.nationalgrid.com/electricity-transmission/document/150571/download

[88] H. of Lords Science and T. Committee, “HOUSE OF LORDS Science and Technology Committee 1st Report of Session 2023-24 Long-duration energy storage: get on with it”, Accessed: Oct. 22, 2024. [Online]. Available: https://members.parliament.uk/members/lords/interests/register-of-lords-interests

[89] S. Schlömer *et al.*, “III ANNEX Technology-specific Cost and Performance Parameters Editor: Lead Authors: Contributing Authors: to the Fifth Assessment Report of the Intergovernmental Panel on Climate Change [Edenhofer Technology-specific Cost and Performance Parameters Annex III AIII Contents,” 2014.

[90] M. J. Haugen, L. Paoli, J. Cullen, D. Cebon, and A. M. Boies, “A fork in the road: Which energy pathway offers the greatest energy efficiency and CO2 reduction potential for low-carbon vehicles?,” *Appl Energy*, vol. 283, p. 116295, Feb. 2021, doi: 10.1016/J.APENERGY.2020.116295.

[91] A. Jahanbakhsh, A. Louis Potapov-Crighton, A. Mosallanezhad, N. Tohidi Kaloorazi, and M. M. Maroto-Valer, “Underground hydrogen storage: A UK perspective,” *Renewable and Sustainable Energy Reviews*, vol. 189, p. 114001, Jan. 2024, doi: 10.1016/J.RSER.2023.114001.

[92] “Stations | UK H2Mobility.” Accessed: Oct. 28, 2024. [Online]. Available: https://www.ukh2mobility.co.uk/stations/

[93] M. K. Jameel *et al.*, “Biogas: Production, properties, applications, economic and challenges: A review,” *Results Chem*, vol. 7, p. 101549, Jan. 2024, doi: 10.1016/J.RECHEM.2024.101549.

[94] “Opportunity areas for district heating networks in the UK National Comprehensive Assessment of the potential for efficient heating and cooling,” 2021.

[95] M. Neuwirth, T. Fleiter, P. Manz, and R. Hofmann, “The future potential hydrogen demand in energy-intensive industries - a site-specific approach applied to Germany,” *Energy Convers Manag*, vol. 252, p. 115052, Jan. 2022, doi: 10.1016/J.ENCONMAN.2021.115052.

[96] “Commercial Air Source Heat Pump or Ground Source Heat Pump UK.” Accessed: Oct. 30, 2024. [Online]. Available: https://www.renewableenergyhub.co.uk/main/heat-pumps-information/commercial-heat-pumps

[97] R. Mutschler, M. Rüdisüli, P. Heer, and S. Eggimann, “Benchmarking cooling and heating energy demands considering climate change, population growth and cooling device uptake,” *Appl Energy*, vol. 288, p. 116636, Apr. 2021, doi: 10.1016/J.APENERGY.2021.116636.
